# Exclusive neuronal detection of KGDHC-specific subunits in the adult human brain cortex despite pancellular protein lysine succinylation

**DOI:** 10.1007/s00429-020-02026-5

**Published:** 2020-01-25

**Authors:** Arpad Dobolyi, Attila Bago, Miklos Palkovits, Natalia S. Nemeria, Frank Jordan, Judit Doczi, Attila Ambrus, Vera Adam-Vizi, Christos Chinopoulos

**Affiliations:** 1 MTA-ELTE Laboratory of Molecular and Systems Neurobiology, Department of Physiology and Neurobiology, Hungarian Academy of Sciences and Eotvos Lorand University, Budapest, 1117 Hungary; 2 Department of Anatomy, Histology and Embryology, Semmelweis University, Budapest, 1094 Hungary; 3 National Institute of Neurosurgery, Budapest, 1145 Hungary; 4 Department of Chemistry, Rutgers University, Newark, NJ 07102-1811 USA; 5 Department of Medical Biochemistry, Semmelweis University, Tuzolto st. 37-47, Budapest, 1094 Hungary; 6 MTA-SE Laboratory for Neurobiochemistry, Semmelweis University, Budapest, 1094 Hungary

**Keywords:** Mitochondria, Succinyl-CoA, Citric acid cycle, Ketoglutarate dehydrogenase

## Abstract

The ketoglutarate dehydrogenase complex (KGDHC) consists of three different subunits encoded by *OGDH* (or *OGDHL*), *DLST,* and *DLD*, combined in different stoichiometries. DLD subunit is shared between KGDHC and pyruvate dehydrogenase complex, branched-chain alpha-keto acid dehydrogenase complex, and the glycine cleavage system. Despite KGDHC’s implication in neurodegenerative diseases, cell-specific localization of its subunits in the adult human brain has never been investigated. Here, we show that immunoreactivity of all known isoforms of OGDHL, OGDH, and DLST was detected exclusively in neurons of surgical human cortical tissue samples identified by their morphology and visualized by double labeling with fluorescent Nissl, while being absent from glia expressing GFAP, Aldhl1, myelin basic protein, Olig2, or IBA1. In contrast, DLD immunoreactivity was evident in both neurons and glia. Specificity of anti-KGDHC subunits antisera was verified by a decrease in staining of siRNA-treated human cancer cell lines directed against the respective coding gene products; furthermore, immunoreactivity of KGDHC subunits in human fibroblasts co-localized > 99% with mitotracker orange, while western blotting of 63 post-mortem brain samples and purified recombinant proteins afforded further assurance regarding antisera monospecificity. KGDHC subunit immunoreactivity correlated with data from the Human Protein Atlas as well as RNA-Seq data from the Allen Brain Atlas corresponding to genes coding for KGDHC components. Protein lysine succinylation, however, was immunohistochemically evident in all cortical cells; this was unexpected, because this posttranslational modification requires succinyl-CoA, the product of KGDHC. In view of the fact that glia of the human brain cortex lack succinate-CoA ligase, an enzyme producing succinyl-CoA when operating in reverse, protein lysine succinylation in these cells must exclusively rely on propionate and/or ketone body metabolism or some other yet to be discovered pathway encompassing succinyl-CoA.

**Electronic supplementary material:**

The online version of this article (10.1007/s00429-020-02026-5) contains supplementary material, which is available to authorized users.

## Introduction

Ketoglutarate dehydrogenase complex (KGDHC) is a multi-subunit enzyme residing in the mitochondrial matrix (Maas and Bisswanger 1990). It catalyzes the irreversible decarboxylation of α-ketoglutarate (αKg) to succinyl-CoA while reducing NAD^+^ to NADH. The complex participates in the citric acid cycle exhibiting a high flux-control coefficient in the generation of reducing equivalents (Cooney et al. 1981; Sheu and Blass 1999), also contributing to the maintenance of the mitochondrial redox state (Gibson et al. 2000). KGDHC’s influence in the cycle is so significant; its activity dictates the directionality of all three of its segments (Chinopoulos 2013). KGDHC is further involved in transaminations and glutamate metabolism (Cooper 2012), while its product, succinyl-CoA, can be shuttled towards heme synthesis (Atamna et al. 2001; Atamna and Frey 2004; Burch et al. 2018), and/or mitochondrial substrate-level phosphorylation (mSLP). mSLP is almost exclusively attributed to succinyl-CoA ligase (SUCL), the enzyme following KGDHC in the citric acid cycle, catalyzing the reversible conversion of succinyl-CoA and ADP (or GDP) to CoASH, succinate, and ATP (or GTP) (Johnson et al. 1998). Unimpaired KGDHC activity is critical for sustaining mSLP (Kiss et al. 2013). Regulation of the complex and its overall impact on energy metabolism has been extensively reviewed elsewhere (Gibson et al. 2010; Starkov 2013).

More recently, the group of Gibson discovered that KGDHC serves as a trans-succinylase, adding succinyl moieties to lysines of proteins in an αKg-dependent manner (Gibson et al. 2015). Lysine succinylation is a widespread posttranslational modification occurring on thousands of lysines residing in hundreds of proteins distributed in the cytosol, nucleus, and mitochondria (Zhang et al. 2011; Weinert et al. 2013).

Mindful of KGDHC’s involvement in a multitude of biochemical processes, it is not surprising that its deficiency leads to pathology, particularly in the brain: indeed, a decline in KGDHC activity is associated with Alzheimer's disease (Gibson et al. 1988, 1998, 2000, 2013; Butterworth and Besnard 1990; Mastrogiacoma et al. 1996; Terwel et al. 1998; Bubber et al. 2005), Parkinson’s disease (Gibson et al. 2003), Huntington’s chorea (Naseri et al. 2015), Wernicke–Korsakoff syndrome (Butterworth et al. 1993), and progressive supranuclear palsy (Albers et al. 2000; Park et al. 2001). A reduction of KGDHC activity is not generally paralleled by all other enzymes of the citric acid cycle (Bubber et al. 2005; Gibson et al. 2010; Bubber et al. 2011). The reason for this selective vulnerability is incompletely understood, but it is likely to be at least partially due to the ability of KGDHC of both producing and affected by reactive oxygen species (Tretter and Adam-Vizi 2004; Starkov et al. 2004), reviewed in (Starkov 2013).

The molecular mechanism(s) linking KGDHC to neurodegeneration are largely unknown; it has been proposed that a decrease in complex activity diminishes glucose uptake and/or metabolism in the brain—a hallmark of Alzheimer’s disease—initiating a sequence of events culminating in neurodegeneration (Chen and Zhong 2013; Sang et al. 2018). Laboratory mice modeling neurodegenerative diseases cross-bread with those exhibiting KGDHC subunit-specific genetic modifications yielded considerable insight regarding pathophysiology (Dumont et al. 2009), but stopped short in offering a breakthrough regarding therapy or even prevention strategy in humans. In most neurodegenerative disorders, specific regions of the brain are involved, and thus, lack of knowledge on KGDHC expression and activity in a cell-specific manner may well underlie the lack of progress regarding identifying suitable therapeutic targets.

Here, we investigated the cell-specific expression of KGDHC subunit components in human cortices obtained from surgeries. KGDHC-specific components were detected in neurons but not glia. However, protein lysine succinylation was pancellularly evident. The latter finding was unexpected, because succinylation requires succinyl-CoA, the product of KGDHC, while glia cannot obtain it from succinate-CoA ligase as they also lack this enzyme (Dobolyi et al. 2015a; b).

## Materials and methods

### Human brains

Human brain samples were collected in accordance to the Ethical Rules for Using Human Tissues for Medical Research in Hungary (HM 34/1999) and the Code of Ethics of the World Medical Association (Declaration of Helsinki). Ethical permission for obtaining surgical brain material for the specific project has been obtained from the Medical Research Council of Hungary (No: 35302-5/2017/EKU), and is valid until June 30th, 2020. Post-mortem tissue samples were taken during brain autopsy in the framework of the Human Brain Tissue Bank (HBTB), Budapest, Hungary. HBTB has been authorized by the Committee of Science and Research Ethic of the Ministry of Health of Hungary (No. 6008/8/2002/ETT) and the Semmelweis University Regional Committee of Science and Research Ethic (No. 32/1992/TUKEB). For autopsy, brains were removed from the skull with a post-mortem delay of 2–6 h. Prior written informed consent was obtained from the patients or from the next of kin for autopsies which included the request to conduct neurochemical analyses. The protocols including analyses of tissue samples were approved by institutional ethics committee of the Semmelweis University. The surgical patients (all males) underwent the removal of brain tumors; samples were obtained from normal brain cortical tissue removed during clearing the path for accessing the area of the tumor. All patients survived the surgery. The subjects whose brains were used in the autopsy study died without any known neurological or affective disorder. The medical history of the subjects was obtained from medical or hospital records, interviews with family members and relatives, as well as from pathological and neuropathological reports. All personal identifiers had been removed and samples were coded before the analyses of tissue. Surgical samples underwent immediate fixation (immersed in ice-cold 4% paraformaldehyde in 0.1 M phosphate-buffered saline within a few seconds after their excision from the brain) for immunolabeling used in histochemical techniques. For western blotting, autopsy samples from 63 different brain regions of 11 subjects (7 females and 4 males) were obtained by microdissection. Individual brain nuclei were microdissected from post-mortem brains (that have been rapidly frozen on dry ice and stored at − 80 °C) using the micropunch technique (Palkovits 1973). Briefly, brains were cut as 1.0–1.5 mm-thick coronal sections, and individual brain regions and nuclei were removed by special punch needles with an inside diameter of 1.0–3.5 mm, using either a head magnifier or a stereomicroscope. The microdissected samples were collected in Eppendorf tubes and stored at − 80 °C until further use. The temperature of brain sections and the microdissected samples was kept under 0 °C during the whole procedure.

### Cell cultures

Fibroblast cultures from skin biopsies were prepared. Cells were grown on poly-l-ornithine coated 25 mm round glass coverslips for 5–7 days, at a density of approximately 8 × 10^5^ cells/coverslip in RPMI1640 medium (GIBCO, Life technologies, Carlsbad, CA, USA) supplemented with 10% fetal bovine serum and 2 mM glutamine and kept at 37 °C in 5% CO_2_. The medium was also supplemented with penicillin, streptomycin, and amphotericin (item A5955, Sigma-Aldrich St. Louis, MO, USA). HeLa cells were grown on poly-l-ornithine coated 25 mm round glass coverslips for 1–2 days, at a density of approximately 5 × 10^5^ cells/coverslip in DMEM (GIBCO) plus glutamine plus 10% fetal calf serum and 1% streptomycin-penicillin.

### Tissue collection for immunolabeling

For immunocytochemistry, brains were cut into 5–10 mm-thick coronal slices and immersion-fixed in 4% paraformaldehyde in 0.1 M phosphate-buffered saline (PBS) for 3–5 days. Subsequently, the blocks were transferred to PBS containing 0.1% sodium azide for 2 days to remove excess paraformaldehyde. Then, the blocks were placed in PBS containing 20% sucrose for 2 days for cryoprotection, after which the blocks were frozen and cut into 50 μm-thick serial coronal sections on a sliding microtome. Sections were collected in PBS containing 0.1% sodium azide and stored at 4 °C until further processing.

### DAB immunolabeling of brain sections

Every fifth free-floating brain section of human temporal and frontal cortical blocks was immunostained for KGDHC subunits. First, the sections were rinsed in PBS containing 0.3% Triton X-100 and 1% bovine serum albumin (BSA) for 1 h to improve antibody penetration and reduce nonspecific labeling, respectively. Subsequently, the antibodies were applied for 48 h at room temperature, followed by incubation of the sections in biotinylated anti-rabbit secondary antibody (1:1,000 dilution; Vector Laboratories, Burlingame, CA) and then in avidin–biotin–peroxidase complex (1:500; Vector Laboratories) for 2 h. Subsequently, the labeling was visualized by incubation in 0.02% 3,3-diaminobenzidine (DAB; Sigma), 0.08% nickel (II) sulfate, and 0.001% hydrogen peroxide in PBS, pH = 7.4 for 5 min. Sections were mounted, dehydrated, and coverslipped with Cytoseal 60 (Stephens Scientific, Riverdale, NJ, USA).

### Double labeling of in brains sections

KGDHC subunits were immunolabeled using fluorescein isothiocyanate (FITC)-tyramide amplification immunohistochemistry, which was followed by labeling neurons and glial cells in a subsequent step. FITC-tyramide amplification immunolabeling of KGDHC subunits was performed as the single labeling except that visualization in the last step was done using fluorescein isothiocyanate (FITC)-tyramide (1:8,000) and H_2_O_2_ in 100 mM Trizma buffer (pH 8.0 adjusted with HCl) applied for 6 min, instead of DAB. Subsequently, sections were placed in mouse anti-glial fibrillary acidic protein (GFAP), a marker of astrocytes (1:300, Santa Cruz Biotechnology, Delaware, CA, USA; see Table 2), rabbit anti-aldehyde dehydrogenase 1 family member L1 (Aldhl1), a marker of astrocytes (1:50, Abcam, Cambridge, UK; see Table 2), rabbit anti-oligodendrocyte transcription factor 2 (Olig2), a marker of oligodendrocytes (1:50, Abcam, Cambridge, UK; see Table 2), mouse anti-myelin basic protein (MBP), a marker of oligodendrocytes (1:300, Abcam, Cambridge, UK; see Table 2), or goat anti-ionized calcium-binding adaptor molecule 1 (Iba1), a marker of microglial cells (1:1000, Abcam, Cambridge, UK; see Table 2) for 48 h at room temperature. The sections were then incubated in Alexa 594 donkey anti-mouse (or rabbit, or goat, respectively) secondary antibody (1:500, Molecular Probes, Eugene, OR) for 2 h and washed. For the double labeling with Nissl staining, the sections were incubated in ‘Neurotrace’ red fluorescent Nissl stain (Molecular Probes) diluted to 1:30 for 2 h, and washed in PBS overnight. Finally, all sections with fluorescent labels were mounted on positively charged slides (Superfrost Plus, Fisher Scientific, Pittsburgh, PA) and coverslipped in antifade medium (Prolong Antifade Kit, Molecular Probes).

### Immunocytochemistry of cell cultures

Fibroblasts or HeLa (CLS Cat# 300194/p772_HeLa, RRID:CVCL_0030) or COS-7 (CLS Cat# 605470/p532_COS-7, RRID:CVCL_0224) cell cultures were first treated with 1 μM Mitotracker Orange (MTO) for 5 min in their culture media, at 37 °C in 5% CO_2_. Subsequent immunocytochemistry of the cultures was performed by fixing the cells with 4% paraformaldehyde in PBS for 20 min, followed by permeabilization by 0.1% TX-100 (in PBS) for 10 min and several washing steps in between with PBS at room temperature. Cultures were treated with 10% donkey serum overnight at 4 °C followed by bathing in 1% donkey serum and a primary antibody as indicated in Tables 1 and 2 for 12 h at room temperature. Titers are indicated in the respective legends. Cells were subsequently decorated using the appropriate Alexa 488-linked or Alexa 647-linked secondary antibody (1:4,000, donkey anti-rabbit, Jackson Immunochemicals Europe Ltd, Cambridgeshire, UK) in the presence of 1% donkey serum.

**Table 1:** Antibodies used for detecting all known human KGDHC subunit isoforms

Subunit	Isoform	Antibody used
OGDHL	Q9ULD0-1	Atlas Antibodies, Cat# HPA052497, RRID:AB_2681853
	Q9ULD0-2	Proteintech Group, Cat# 17110-1-AP, RRID:AB_2156767
	Q9ULD0-3	Proteintech Group, Cat# 17110-1-AP, RRID:AB_2156767
OGDH	Q02218-1	Proteintech Group, Cat# 15212-1-AP, RRID:AB_2156759
	Q02218-2	Proteintech Group, Cat# 15212-1-AP, RRID:AB_2156759
	Q02218-3	Atlas Antibodies, Cat# HPA020347, RRID:AB_1854773
DLST	P36957-1	Cell Signaling Technology, Cat# 11954, RRID:AB_2732907
	P36957-2	Cell Signaling Technology, Cat# 11954, RRID:AB_2732907
DLD	P09622-1	Abcam, Cat# ab133551, RRID:AB_2732908
	P09622-2	Abcam, Cat# ab133551, RRID:AB_2732908
	P09622-3	Abcam, Cat# ab133551, RRID:AB_2732908

**Table 2:** Antibodies directed against other targets used in this study

Target	Antibody used
GFAP	Santa Cruz Biotech, Cat# sc-33673, RRID:AB_627673
Aldhl1	Abcam, Cat# ab177463, RRID: AB_2811300
Myelin basic protein	Abcam, Cat# ab24567, RRID:AB_448144
Olig2	Abcam, Cat# ab109186, RRID: AB_10861310
IBA1	Abcam, Cat# ab107159, RRID:AB_10972670
Succinyl-lysine	NovoPro, Cat# 106768, RRID:AB_2732922
VDAC1	Abcam, Cat# ab154856, RRID:AB_2687466
SUCLG1	Abcam, Cat# ab97867, RRID:AB_10678848
SUCLG2	Atlas Antibodies, Cat# HPA046705, RRID:AB_2679762
SUCLA2	Proteintech Group, Cat# 12627-1-AP, RRID:AB_2271200
β-actin	Abcam, Cat# ab6276, RRID:AB_2223210

### Western blotting

Frozen brain samples were thawn on ice in the presence of radioimmunoprecipitation assay buffer and a protease cocktail inhibitor containing: 0.5 mM 4-(2-aminoethyl) benzenesulfonyl fluoride hydrochloride, 150 nM Aprotinin, 1 μM E-64, 0.5 mM EDTA disodium, and 1 μM Leupeptin, and homogenized with a Teflon pestle. The suspensions were centrifuged once at 10,000*g* for 10 min, and the proteins present in the supernatants were separated by sodium dodecyl sulfate—polyacrylamide gel electrophoresis (SDS-PAGE). Separated proteins were transferred to a methanol-activated polyvinylidene difluoride membrane. Immunoblotting was performed as recommended by the manufacturers of the antibodies, see Tables 1 and 2. Immunoreactivity was detected using the appropriate peroxidase-linked secondary antibody (1:4,000, donkey anti-mouse or anti-rabbit, Jackson Immunochemicals Europe Ltd, Cambridgeshire, UK) and enhanced chemiluminescence detection reagent (ECL system; Amersham Biosciences GE Healthcare Europe GmbH, Vienna, Austria).

### Image processing

The sections were examined using an Olympus BX60 light microscope equipped with bright-field, dark-field and fluorescence. Images were captured at 2048 × 2048 pixel resolution with an SPOT Xplorer digital CCD camera (Diagnostic Instruments, Sterling Heights, MI, USA) using a 4 × objective for dark-field images, and 4–40 × objectives for bright-field and fluorescent images. Fluorescent sections were also evaluated using a Bio-Rad 2100 Rainbow Confocal System (Bio-Rad Laboratories, Inc, CA, USA). The contrast and sharpness of the images were adjusted using the “levels” and “sharpness” commands in Adobe Photoshop CS 8.0. Full resolution was maintained until the photomicrographs were finally cropped at which point the images were adjusted to a resolution of 300 dpi.

### siRNA and cell transfections

The ON-TARGETplus SMARTpool containing four different siRNA sequences, all specific to human KGDHC-specific components (see under “Results”) and the corresponding non-targeting control (scrambled RNA), were designed by Thermo Scientific Dharmacon and synthesized by Sigma-Aldrich. HeLa cells were transfected with 100 nM of either siRNA or scrambled siRNA using Lipofectamine 2000 according to the manufacturer’s instructions, 48 h before immunocytochemistry.

## Results

### Antibody selection for detecting all known KGDHC subunit human isoforms

KGDHC consists of multiple copies of three subunits: oxoglutarate dehydrogenase (OGDH) or oxoglutarate dehydrogenase-like protein (OGDHL), dihydrolipoyl succinyltransferase (DLST), and dihydrolipoyl dehydrogenase (DLD). OGDHL exhibits three isoforms Q9ULD0-1, Q9ULD0-2 and Q9ULD0-3; OGDH 3 isoforms: Q02218-1, Q02218-2 and Q02218-3; DLST 2 isoforms: P36957-1 and P36957-2; and DLD 3 isoforms: P09622-1, P09622-2 and P09622-3. By knowing the amino acid sequence of each isoform, we could select antibodies raised using epitopes recognizing all isoforms, see Table 1. Whenever the same antibody is used for more than one isoform, this is because the epitope is within a 100% aligning region between the isoforms. More antibodies were probed that yielded no staining and these were excluded from this study.

### Antibody validation

Antibodies directed against KGDHC subunit isoforms were validated by the following protocols: (1) > 99% co-localization with mitotracker orange (a dye that stains exclusively mitochondria) in human fibroblasts; (2) decrease in immunocytochemical staining of siRNA—but not scramble RNA-treated human cell lines silencing genes that code KGDHC subunit isoforms and decorated by the same antibodies; (3) emergence of only one band at the expected molecular weight in Western blots probing purified, recombinant proteins, and human brain homogenate samples.

**Fig. 1:**
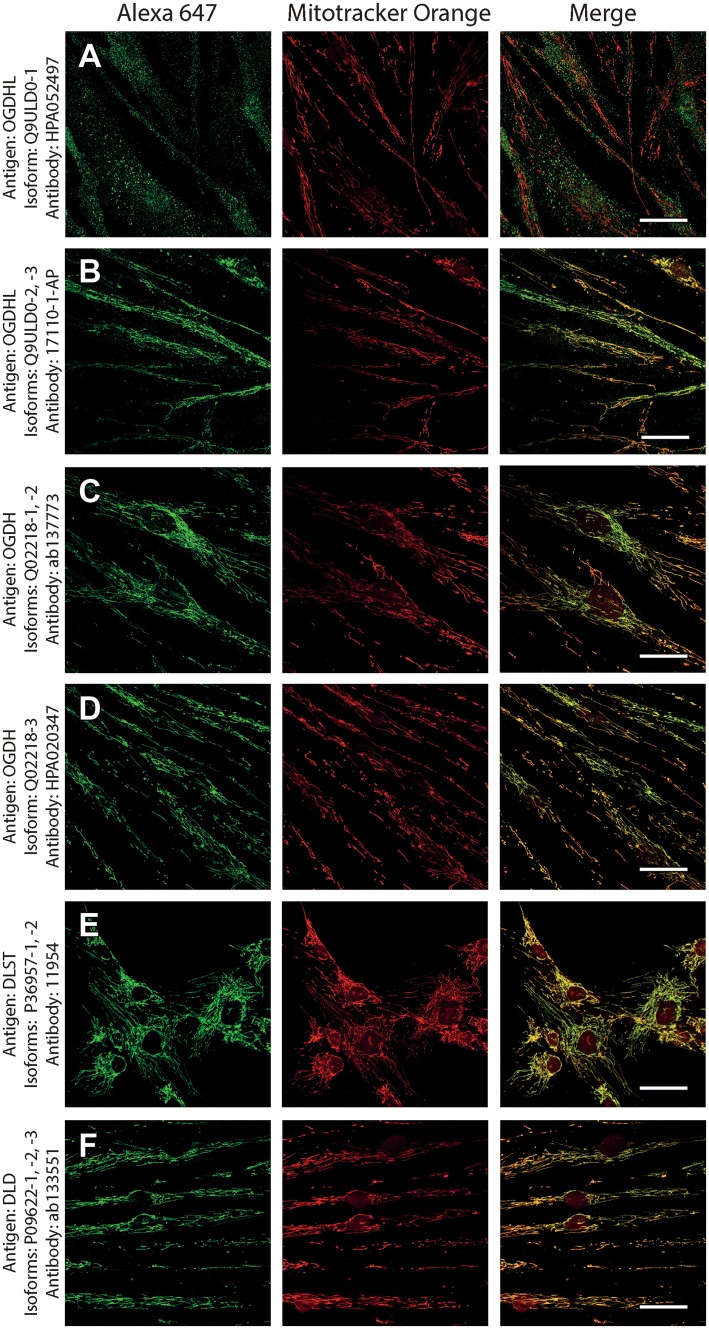
The demonstration of mitochondrial localization of OGDHL, OGDH, DLST, and DLD in human fibroblasts using the antibodies indicated on the left. OGDHL (**a**, **b**), OGDH (**c**, **d**), DLST (**e**), and DLD (**f**) immunolabeling (labelling by Alexa 647) in human fibroblasts in relation to mitotracker orange (MTO). Scale bars = 30 µm for **a** and **c**, and 50 µm for **b, d**–**f**

As shown in Fig. 1, normal human fibroblasts were treated with the antibodies indicated on the left and detected with secondary antibodies conjugated with Alexa 647 fluorophore (left panels, green); their mitochondrial network was selectively stained by loading cells with Mitotracker Orange (MTO, 1 μM, middle panels, red) prior to fixation. Co-localization of Alexa 647 and MTO staining is shown in the panels to the right. From the right-hand panels, it is evident that except for antibody HPA052497 directed against isoform 1 of OGDHL (Q9ULD0-1), all other antibodies yielded > 99% of co-localization with the mitochondrial network. Regarding Q9ULD0-1, at this junction, it cannot be distinguished if the lack of co-localization of the antibody with MTO is due to lack of specificity, or Q9ULD0-1 is not sufficiently expressed in human fibroblasts. Nonetheless, the robust co-localization of all other antibodies with MTO in these confocal images proved that the antigens are located within mitochondria.

**Fig. 2:**
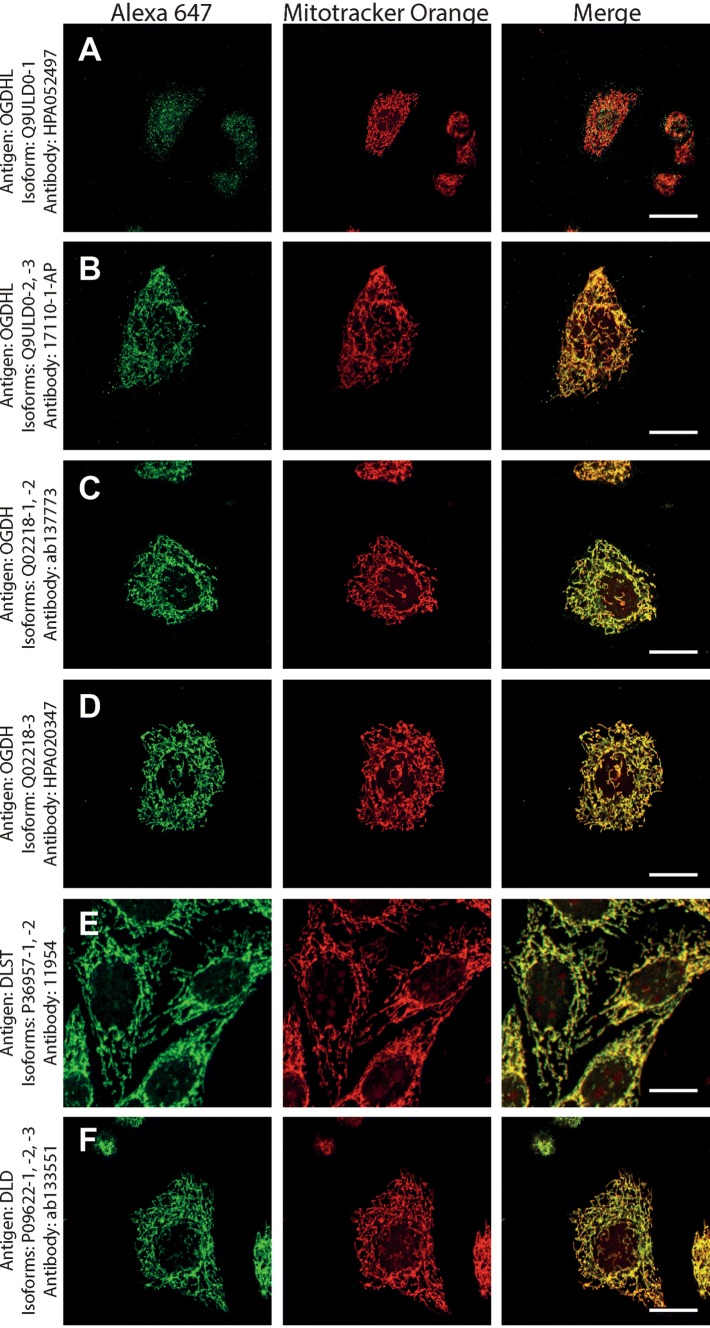
The demonstration of mitochondrial localization of OGDHL, OGDH, DLST, and DLD in HeLa cells using the antibodies indicated on the left. OGDHL (**a**, **b**), OGDH (**c**, **d**), DLST (**e**), and DLD (**f**) immunolabeling (labelling by Alexa 647) in HeLa cells in relation to mitotracker orange (MTO). Scale bars = 30 µm for **a** and 15 µm for **b**–**f**

**Fig. 3:**
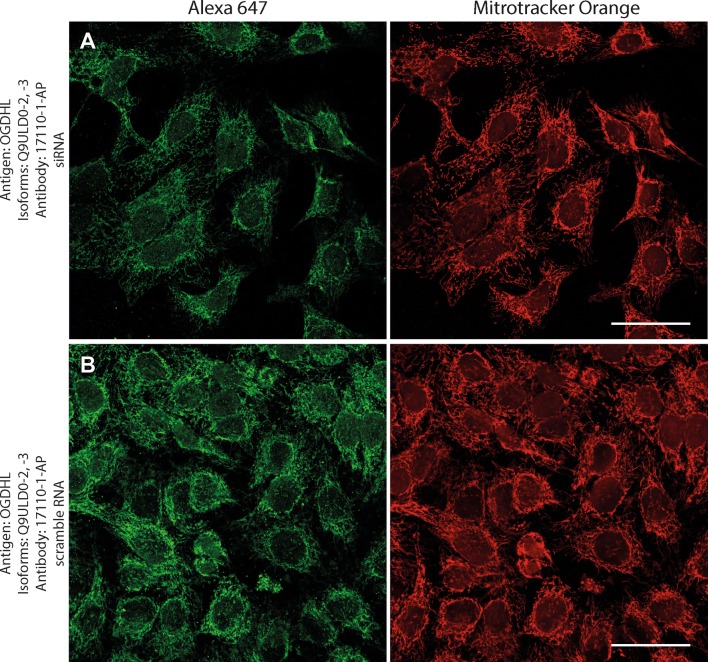
Effect of siRNA (**a**) vs scramble RNA (**b**) directed against OGDHL isoforms 2 and 3 on OGDHL immunostaining in HeLa cells in relation to mitotracker orange (MTO). A reduced intensity of OGDHL immunolabeling is visible following siRNA treatment as compared to scramble RNA treatment, while siRNA has no effect on the labeling with MTO. Scale bars = 50 µm

**Fig. 4:**
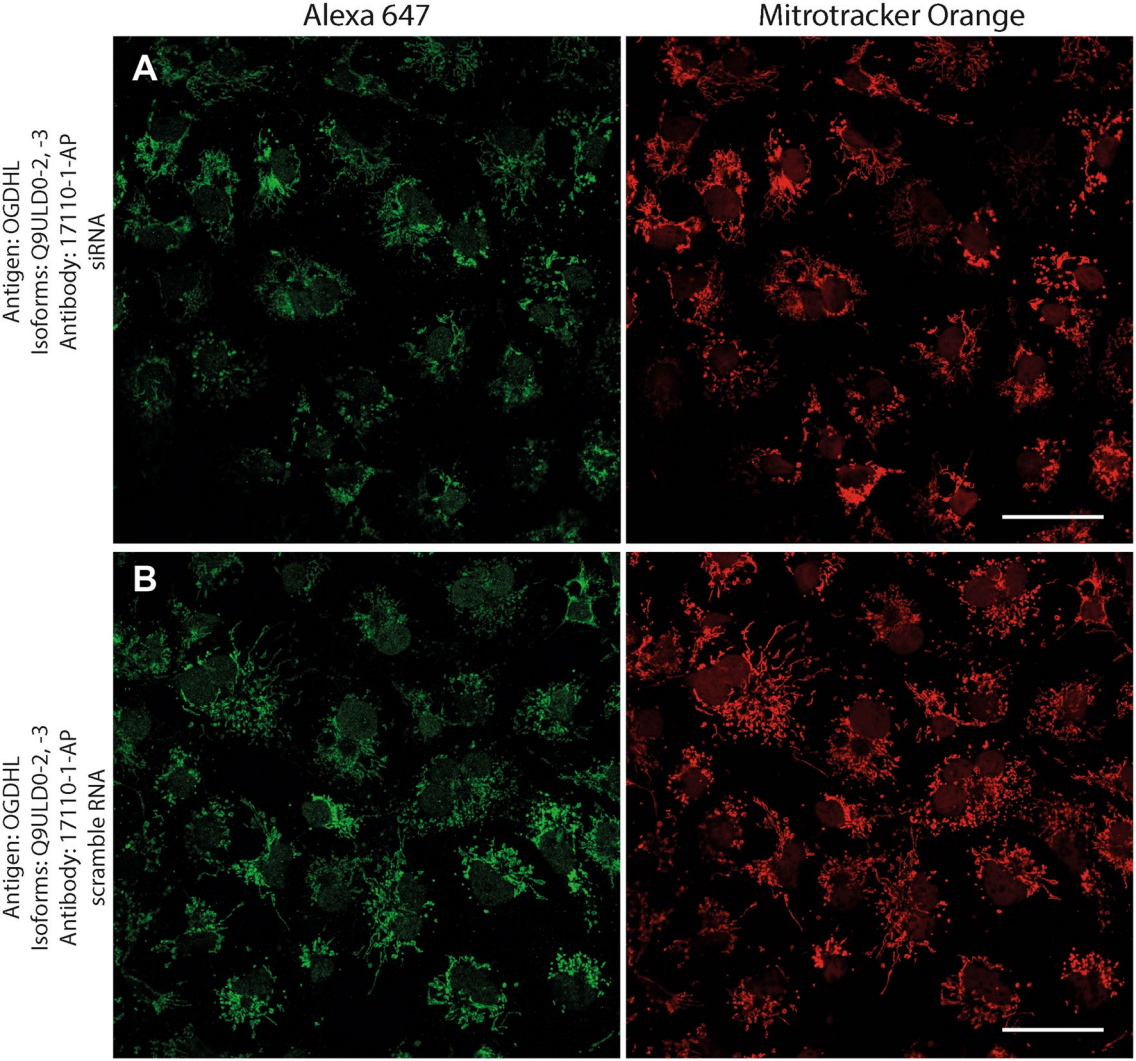
Effect of siRNA (**a**) vs scramble RNA (**b**) directed against OGDHL isoforms 2 and 3 on OGDHL immunostaining in COS-7 cells in relation to mitotracker orange (MTO). A reduced intensity of OGDHL immunolabeling is visible following siRNA treatment as compared to scramble RNA treatment, while siRNA has no effect on the labeling with MTO. Scale bars = 50 µm

**Fig. 5:**
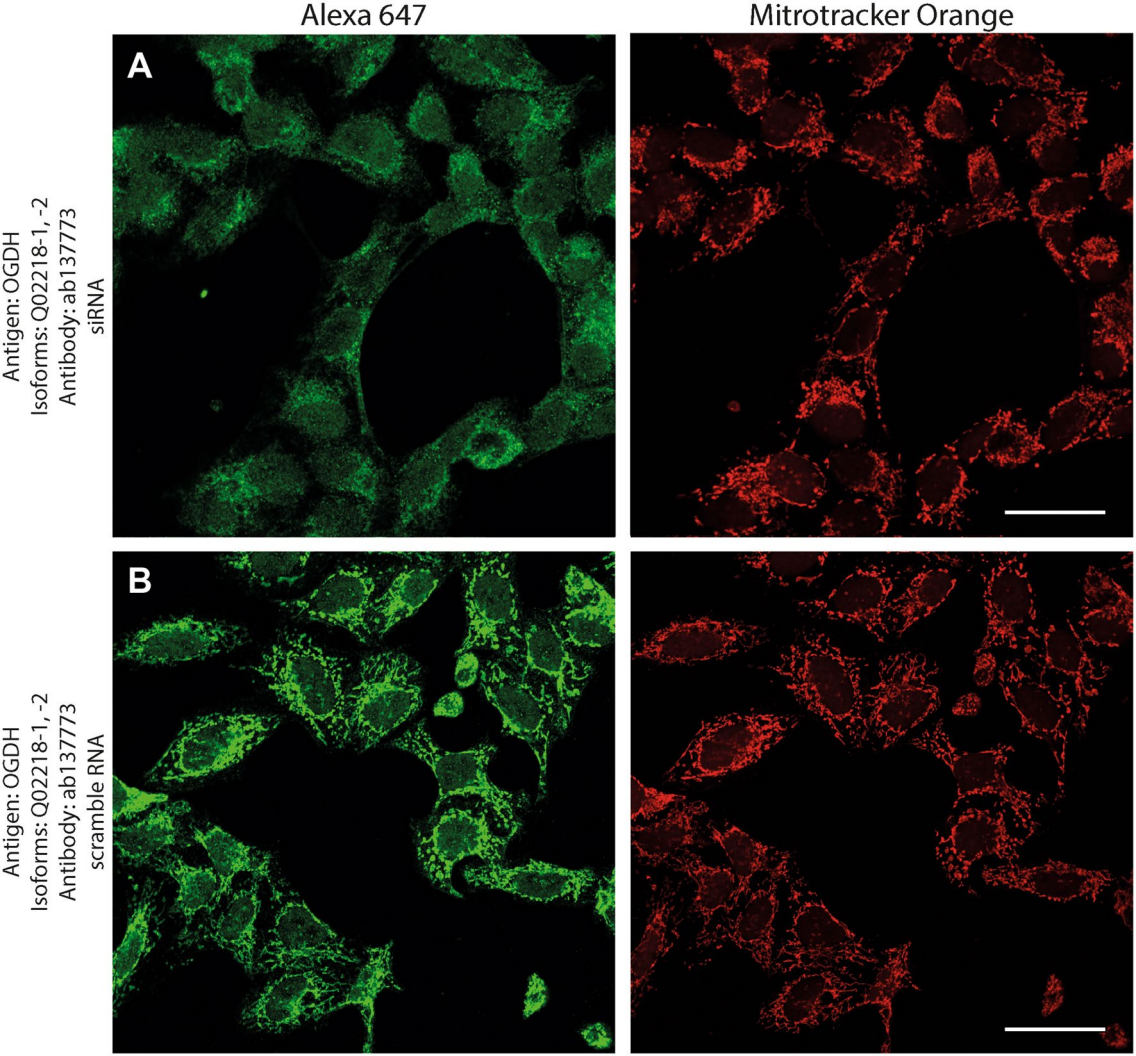
Effect of siRNA (**a**) vs scramble RNA (**b**) directed against OGDH isoforms 1 and 2 on OGDH immunostaining in HeLa cells in relation to mitotracker orange (MTO). A reduced intensity of OGDH immunolabeling is visible following siRNA treatment as compared to scramble RNA treatment, while siRNA has no effect on the labeling with MTO. Scale bars = 50 µm

**Fig. 6:**
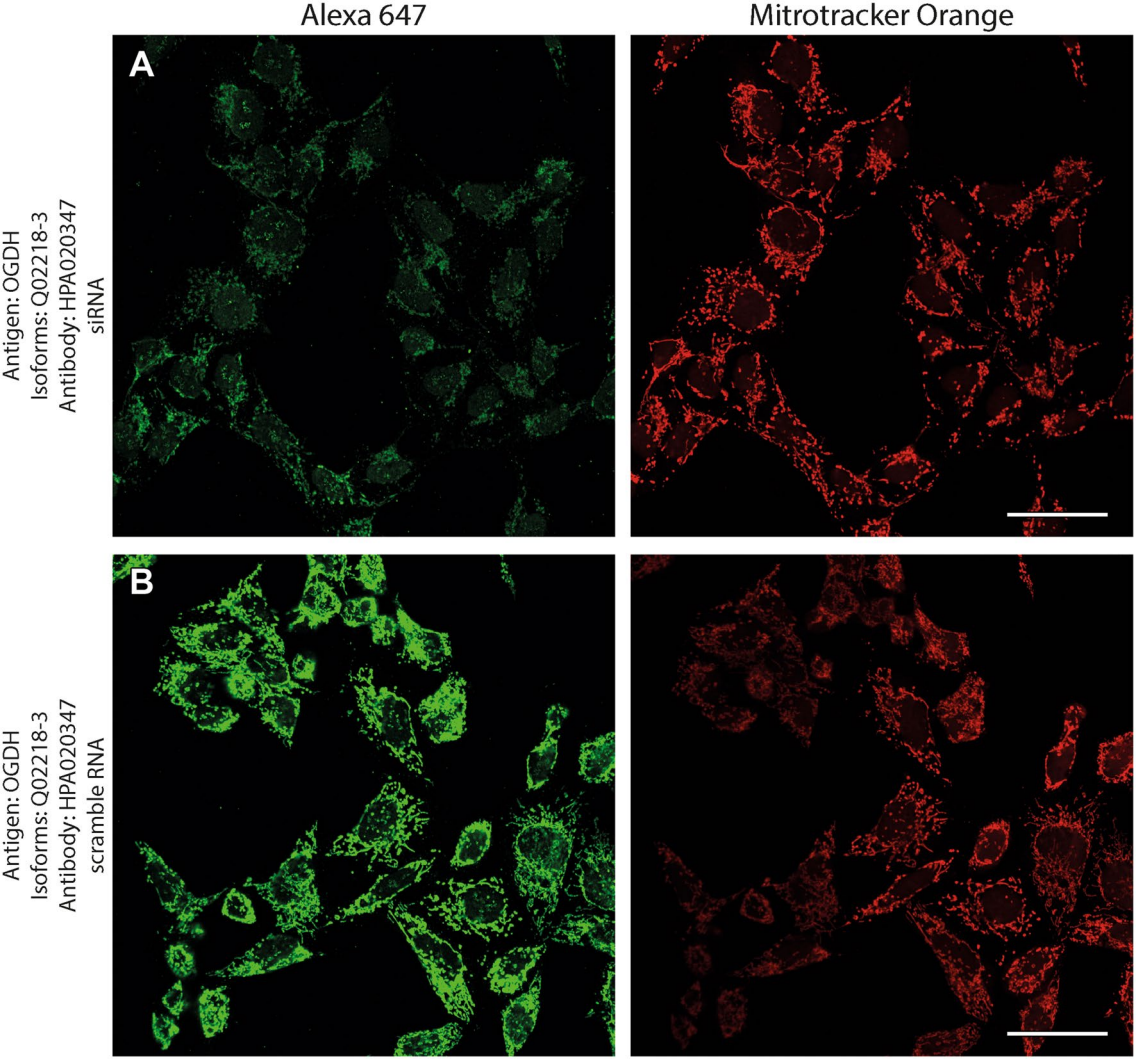
Effect of siRNA (**a**) vs scramble RNA (**b**) directed against OGDH isoform 3 on OGDH immunostaining in HeLa cells in relation to mitotracker orange (MTO). A reduced intensity of OGDH immunolabeling is visible following siRNA treatment as compared to scramble RNA treatment, while siRNA has no effect on the labeling with MTO. Scale bars = 50 µm

**Fig. 7:**
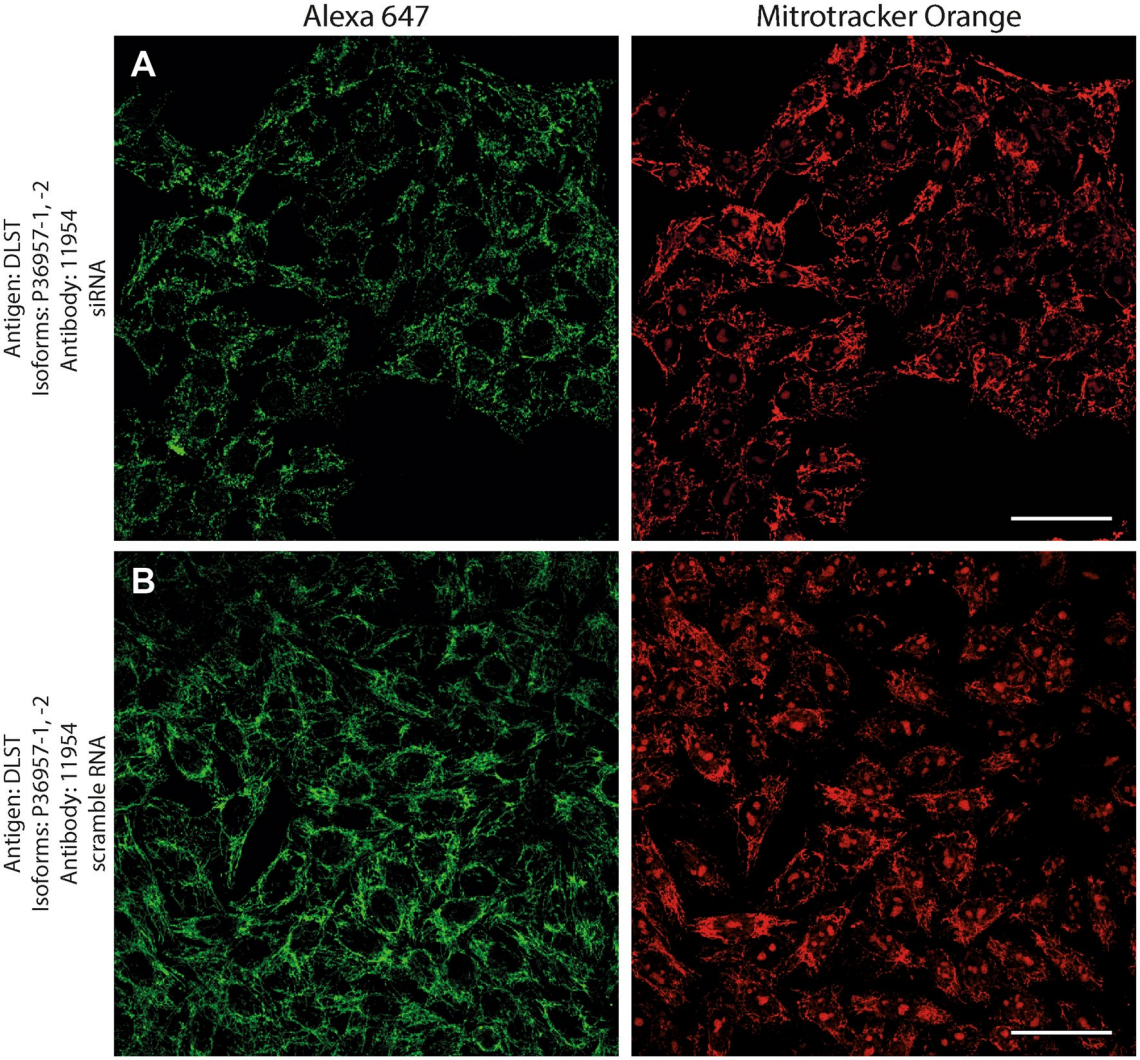
Effect of siRNA (**a**) vs scramble RNA (**b**) directed against DLST isoforms 1 and 2 on DLST immunostaining in HeLa cells in relation to mitotracker orange (MTO). A reduced intensity of DLST immunolabeling is visible following siRNA treatment as compared to scramble RNA treatment, while siRNA has no effect on the labeling with MTO. Scale bars = 50 µm

**Fig. 8:**
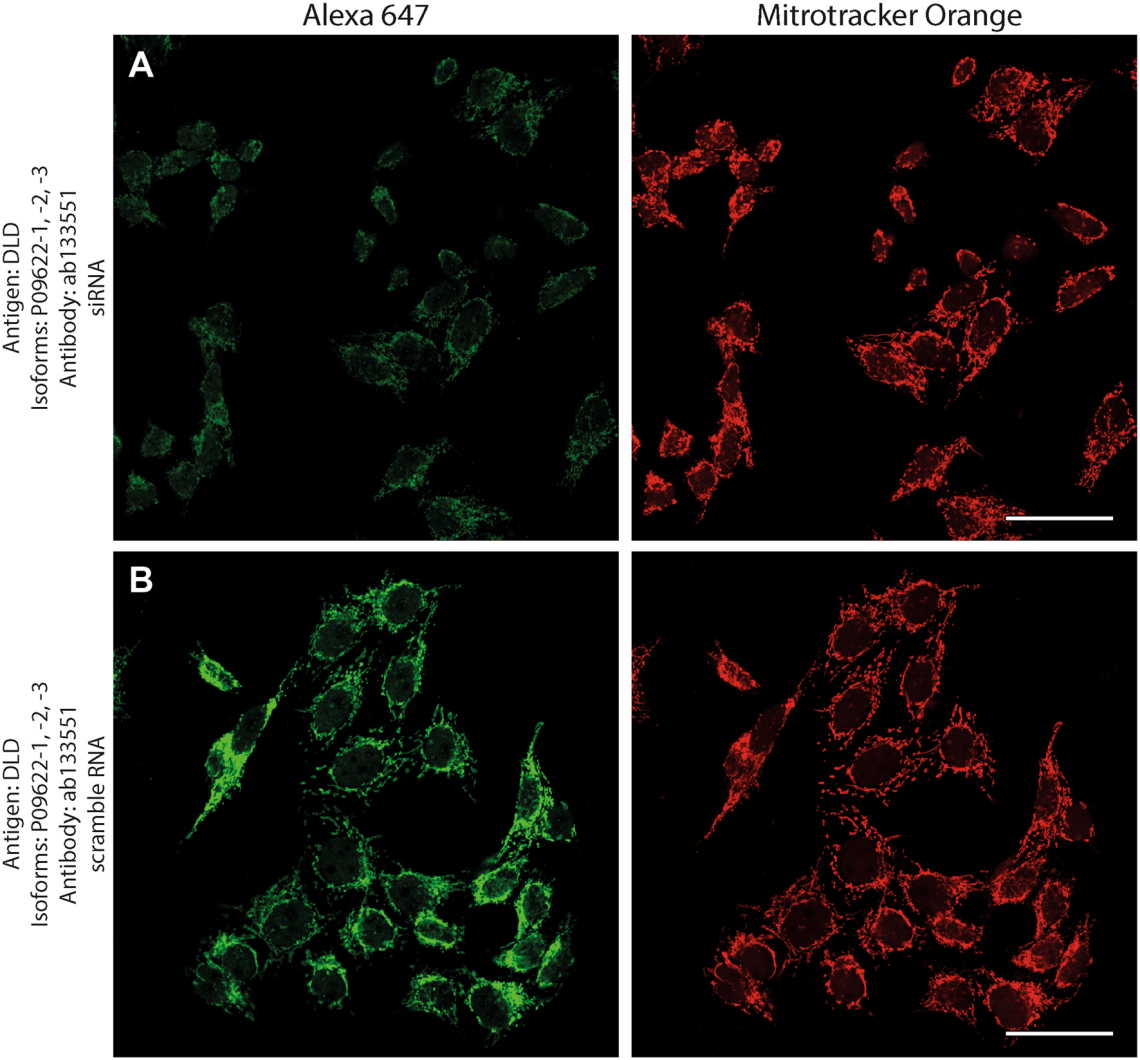
Effect of siRNA (**a**) vs scramble RNA (**b**) directed against DLD isoforms 1, 2 and 3 on DLD immunostaining in HeLa cells in relation to mitotracker orange (MTO). A reduced intensity of DLD immunolabeling is visible following siRNA treatment as compared to scramble RNA treatment, while siRNA has no effect on the labeling with MTO. Scale bars = 50 µm

Next, to investigate if the intramitochondrial decoration is due to antigens belonging to the intended proteins against which the KGDHC subunit and isoform-specific antibodies were raised, cell lines were transfected with either siRNA directed against individual subunits belonging to KGDHC or scramble RNA, and subsequently co-stained with the same antibodies and MTO. For these experiments, cancer cell lines (HeLa and COS-7) were used instead of fibroblasts, because the former exhibit much higher transfection efficiencies than the latter. COS-7 is a cell line originating from monkey kidney tissue, but it was probed for OGDHL isoforms 2 and 3 which are identical to those expressed in humans. Other cell lines tested did not yield a sufficiently clear mitochondrial network for co-localization studies (not shown). As shown in Fig. 2, HeLa cells were treated with the antibodies indicated on the left and decorated with secondary antibodies conjugated with Alexa 647 fluorophore (left panels, green); their mitochondrial network was selectively stained by loading cells with MTO (1 μM, middle panels, red) prior to fixation. Co-localization of Alexa 647 and MTO staining is shown in the panels to the right. The robust co-localization of all antibodies (except HPA052497) with MTO in these confocal images proved that the antigens are located within mitochondria of HeLa cells as well. As shown in the top rows of Figs. 3, 4, 5, 6, 7, and 8, cells were treated with siRNA directed against the respective protein, and in the bottom rows with scramble RNA. Alexa 647 decoration is shown in panels to the left, while MTO staining is shown in the panels to the right. It is evident that the mitochondrial staining for KGDHC subunits shown in siRNA-treated cells (top-left images) appears weaker than the scramble RNA-treated cells (bottom-left panels); furthermore, in some instances (top left panels in Figs. 5, 6, and 8), the mitochondrial network of siRNA-treated cells is more fragmented than those treated with the scramble RNA. The decrease in staining together with the alterations in mitochondrial network in siRNA-treated but not scramble RNA-treated cells strongly argues that the antibodies used are specific for the intended KGDHC subunit proteins. It must be noted that in some panels, immunoreactivity with siRNA may appear similar to immunoreactivity without any intervention; however, by semi-quantitating emitted light intensity of the signal minus the background, immunoreactivity presented for si RNA is consistently lower than that for scramble RNA (not shown).

**Fig. 9:**
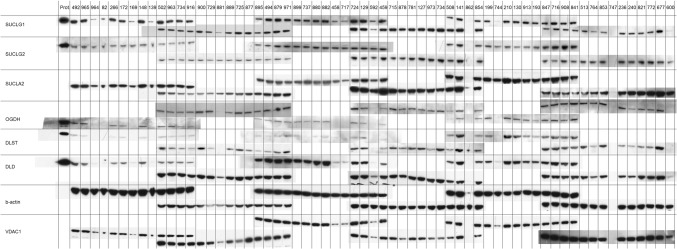
Immunoreactivities of the proteins indicated on the left in brain tissue homogenates (indicated on the top; individual samples coded by a three-digit number obtained from brain regions as detailed in Table 3). The most-left lane includes purified, recombinant proteins (Prot.) for SUCLG1, SUCLG2, OGDH, DLST, and DLD

Subsequently, to afford further assurance that the antibodies used exhibit monospecificity for the intended proteins -which cannot be addressed by immunocytochemistry—and they yield reproducible results from a sufficiently high number of tissues, we performed western blotting analysis on purified, recombinant proteins (Nemeria et al. 2014; Ambrus et al. 2016) and 63 human brain homogenates obtained from 11 cadaveric brain donors. All antibodies are directed against short stretches of amino acid sequences (which was important for selecting isoform-specific antibodies), and thus, they were expected to perform equally well in western blotting protocols where protein folding is lost due to the presence of strong detergents. Antibody-selling company policies prohibit the publication of the epitopes (to which the authors disagree). As shown in Fig. 9, recombinant proteins (shown on the left-most lane) and the brain homogenates were probed for SUCLG1, SUCLG2, SUCLA2, OGDH, DLST, DLD, β-actin, and VDAC1. Antibodies directed against OGDHL isoforms were not suitable for western blotting. Since the number of the homogenates used was higher than the number of lanes per gel that could be loaded, samples loaded in the last four lanes of each gel was also loaded in the first four lanes of the next gel. β-actin and VDAC1 served as loading markers for total tissue homogenate and mitochondrial content, respectively. SUCLG1, SUCLG2, and SUCLA2 correspond to subunits of succinate-CoA ligase, the enzyme following KGDHC in the citric acid cycle. On the top lane, each three-digit number represents a brain homogenate sample from a brain donor. The exact brain location from where these homogenates originated is shown in Table 3. As shown in the cropped scanned western blots, the antibodies raised against OGDH, DLST, and DLD yielded a band which appeared at the same molecular weight as the recombinant protein. Antibodies for SUCLG1, SUCLG2, and SUCLA2 have been validated elsewhere (Dobolyi et al. 2015a; b; Chinopoulos et al. 2018). Scanned images of whole blots appear in the supplemental material. In supplemental Fig. 1, it is shown that antibodies yielded—in the vast majority of cases—a single band at the expected molecular weight; when the blots were “overexposed”, several new bands appeared that were much fainter than the band corresponding to the intended protein. From the results shown in western blots and confocal imaging of immunocytochemistry, we concluded that the antibodies raised against KGDHC components are specific to the intended proteins. However, it must be emphasized that this did not apply for those raised against OGDHL isoforms.

**Table 3:** Brain regions of origin of samples

Sample identifier	Brain region
492	Insular cortex
965	putamen
964	caudate nucleus
82	Parahippocampal cortex
266	Entorhinal cortex
172	Somatosensory cortex
169	Cerebral white matter
148	Insular cortex
139	Cerebral white matter
502	Temporal cortex
963	Insular cortex
734	Posterior cingulate cortex
916	Parahippocampal cortex
900	Somatosensory cortex
729	Parahippocampal cortex
881	Cerebral white matter
889	Somatomotor cortex
725	Insular cortex
877	Dorsomedial prefrontal cortex
895	Temporal cortex
494	Anterior cingulate cortex
879	Frontopolar cortex
971	Frontal cortex
899	Caudal cingulate cortex
737	Entorhinal cortex
880	Parahippocampal cortex
882	Frontal cortex
458	Cerebral white matter (frontal)
717	Cerebral white matter (frontal)
724	Temporal cortex
129	Somatomotor cortex
592	Cerebral white matter (frontal)
459	Frontal cortex
715	Frontal cortex
878	Ventrolateral prefrontal cortex
781	Posterior cingulate cortex
127	Somatosensory cortex
973	Temporal cortex
734	Posterior cingulate cortex
508	Posterior cingulate cortex
141	Cerebral white matter (frontal)
862	Posterior cingulate cortex
854	Entorhinal cortex
199	Anterior cingulate cortex
744	Cerebral white matter (frontal)
210	Temporal cortex
130	Temporal cortex
913	medial geniculate body
193	Frontal cortex
847	Anterior cingulate cortex
716	Anterior cingulate cortex
908	Frontal cortex
841	Insular cortex
513	Posterior cingulate cortex
764	Cerebral white matter (frontal)
853	Temporal cortex
747	Frontal cortex
236	Insular cortex
240	Parahippocampal cortex
821	Parahippocampal cortex
772	Temporal cortex
677	Entorhinal cortex
600	Cerebral white matter (frontal)

### KGDHC subunit- and isoform-specific immunoreactivity in human brain cortex and cell types identification by co-staining with neuronal and/or glial markers

**Fig. 10:**
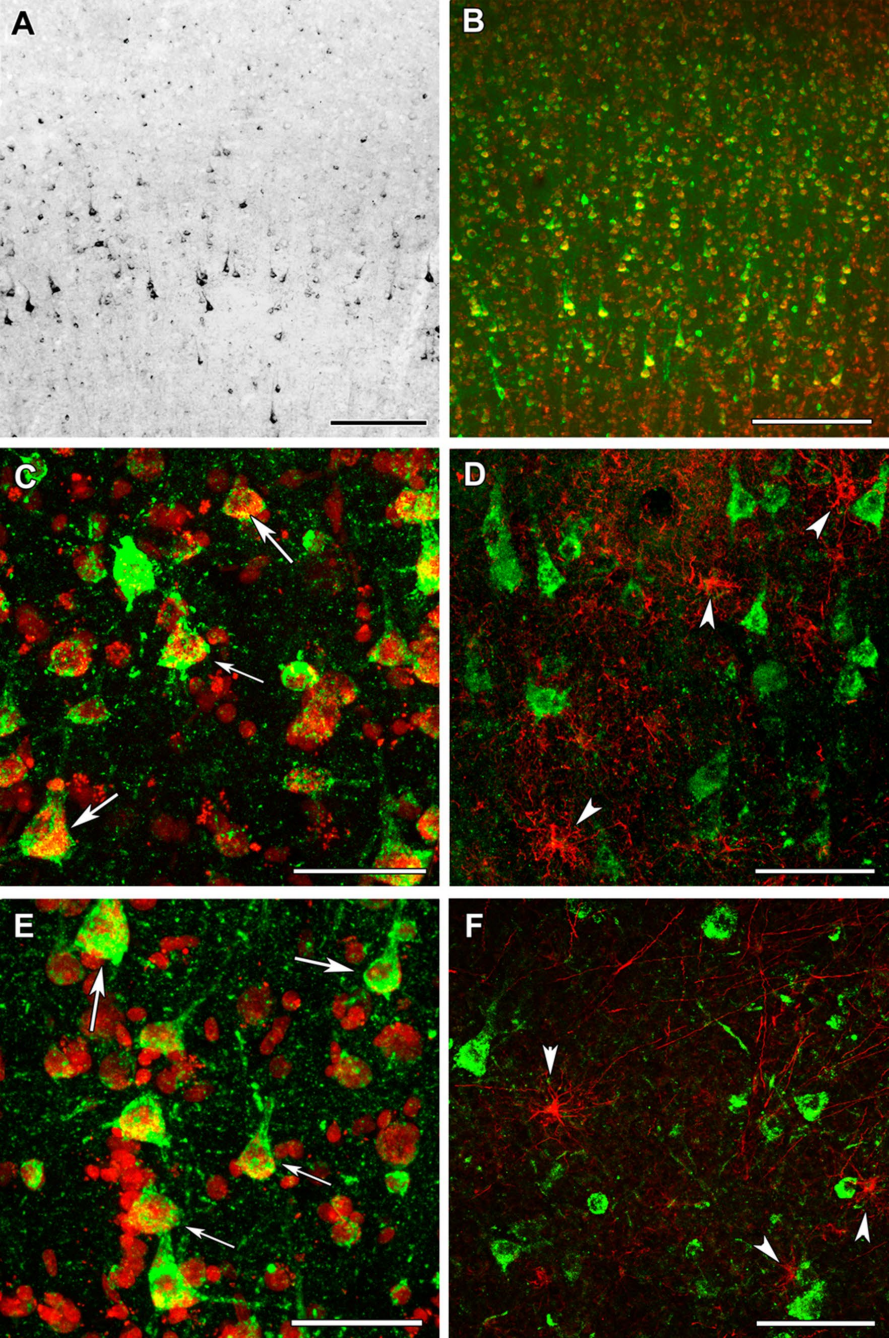
OGDHL immunolabeling in the human cerebral cortex in relation to neuronal and glial markers. **a** OGDHL isoform 1-immunoreactive cells are present the cerebral cortex, with higher density in the deep layers. **b** OGDHL isoform 1 (green) and fluorescent Nissl staining (red) show many double-labeled yellow cells in the cerebral cortex. **c** A higher magnification confocal image of a cerebral cortical section double stained with OGDHL isoform 1 (green) and fluorescent Nissl staining (red) demonstrates that larger, neuronal cells (some of them indicated with white arrow) are double labeled. Several small cells are also present, which are not labeled with OGDHL isoform 1. Please observe the dot-like intracellular labeling pattern of OGDHL subunit 1 immunoreactivity. **d** A cerebral cortical section double labeled with OGDHL isoform 1 (green) and the established astrocyte marker glial fibrillary acidic protein (GFAP; red) shows only single-labeled cells positive for OGDHL isoform 1 or for GFAP, demonstrating that astrocytes are not labeled with OGDHL isoform 1. Some of the single-labeled astrocytes are pointed to by white arrowheads. **e** A confocal image of a cerebral cortical section double stained with OGDHL isoform 2/3 (green) and fluorescent Nissl staining (red) demonstrates that larger, neuronal cells (some of them indicated with white arrow) are double labeled. Several small cells not labeled with OGDHL isoform 2/3 are also present. Note the dot-like intracellular labeling pattern of OGDHL subunit 2/3 immunoreactivity. **f** A cerebral cortical section double labeled with OGDHL isoform 2/3 (green) and GFAP (red) shows cells labeled either with OGDHL or with GFAP, demonstrating that astrocytes are not labeled with OGDHL isoform 2/3. Some of the single-labeled astrocytes are pointed to by white arrowheads. Scale bars = 200 µm for **a**, 300 µm for **b**, 100 µm for **c**–**f**

**Fig. 11:**
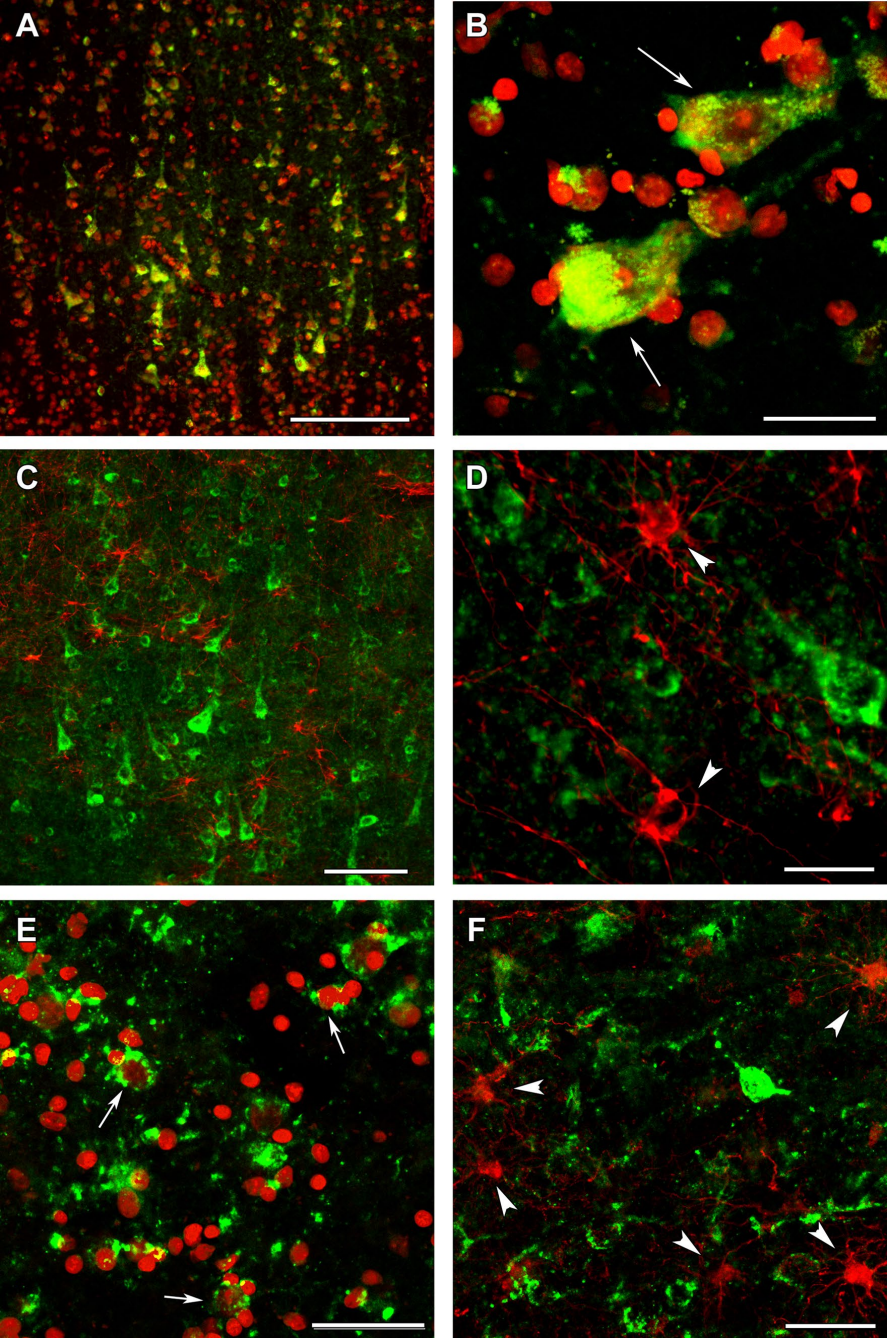
OGDH immunolabeling in the human cerebral cortex in relation to neuronal and glial markers. **a** OGDH isoform 1/2 (green) immunolabeling and fluorescent Nissl staining (red) show many double-labeled yellow cells in the cerebral cortex. **b** A high magnification confocal image of a cerebral cortical section double stained with OGDH isoform 1/2 (green) and fluorescent Nissl staining (red) demonstrates that larger, neuronal cells indicated with white arrow are double labeled. Several small cells are also present, which are not labeled with OGDH isoform 1/2. Note the dot-like intracellular labeling pattern of OGDH subunit 1/2 immunoreactivity. **c** A cerebral cortical section double labeled with OGDH isoform 1/2 (green) and the established astrocyte marker glial fibrillary acidic protein (GFAP; red) shows only single-labeled cells positive for OGDH isoform 1/2 or for GFAP, demonstrating that astrocytes are not labeled with OGDH isoform 1. **d** A high magnification confocal image shows separate single OGDH isoform 1/2 and GFAP-positive (red, pointed to by arrowheads) cells. **e** A confocal image of a cerebral cortical section double stained with OGDH isoform 3 (green) and fluorescent Nissl staining (red) demonstrates that larger, neuronal cells (some of them indicated with white arrow) are double labeled. Several small cells not labeled with OGDH isoform 3 are also present. **f** A cerebral cortical section double labeled with OGDH isoform 3 (green) and GFAP (red) shows cells labeled either with OGDH isoform 3 or with GFAP, demonstrating that astrocytes are not labeled with OGDH isoform 3. Some of the single-labeled astrocytes are pointed to by white arrowheads. Scale bars = 200 µm for **a**, 30 µm for **b**, 100 µm for **c**, 25 µm for **d**, and 50 µm for **e** and **f**

Using the above validated antibodies, we performed immunohistochemistry in human cerebral cortical material obtained from neurosurgical interventions. Frontal and temporal cortical samples did not show any visible difference in their labeling pattern for any antibody. The distribution of OGDH subunits investigated with antibodies specific to different OGDHL and OGDH isoforms was similar (Figs. 10 and 11, respectively). Labeled cells were scarce in layer I of the cerebral cortex (Fig. 10a). In contrast, a high number of labeled cells were present in all other layers of the cerebral cortex (Fig. 10a and 11a). The labeling was absent in the nuclei of the cells, while it was punctate with occasional filamentous appearance in the perikarya. Although in situ* mitochondria* are filamentous, a largely punctate appearance emerged likely because of the multiple amplification steps. Furthermore, immunohistochemistry protocols of signal amplification almost always lead to diminished spatial resolution. Thus, KGDHC staining in the human cerebral cortical tissue did not completely match the filamentous mitochondrial network commonly observed in cultured fibroblasts and cell lines, but its appearance was still indicative of mitochondrial localization.

**Fig. 12:**
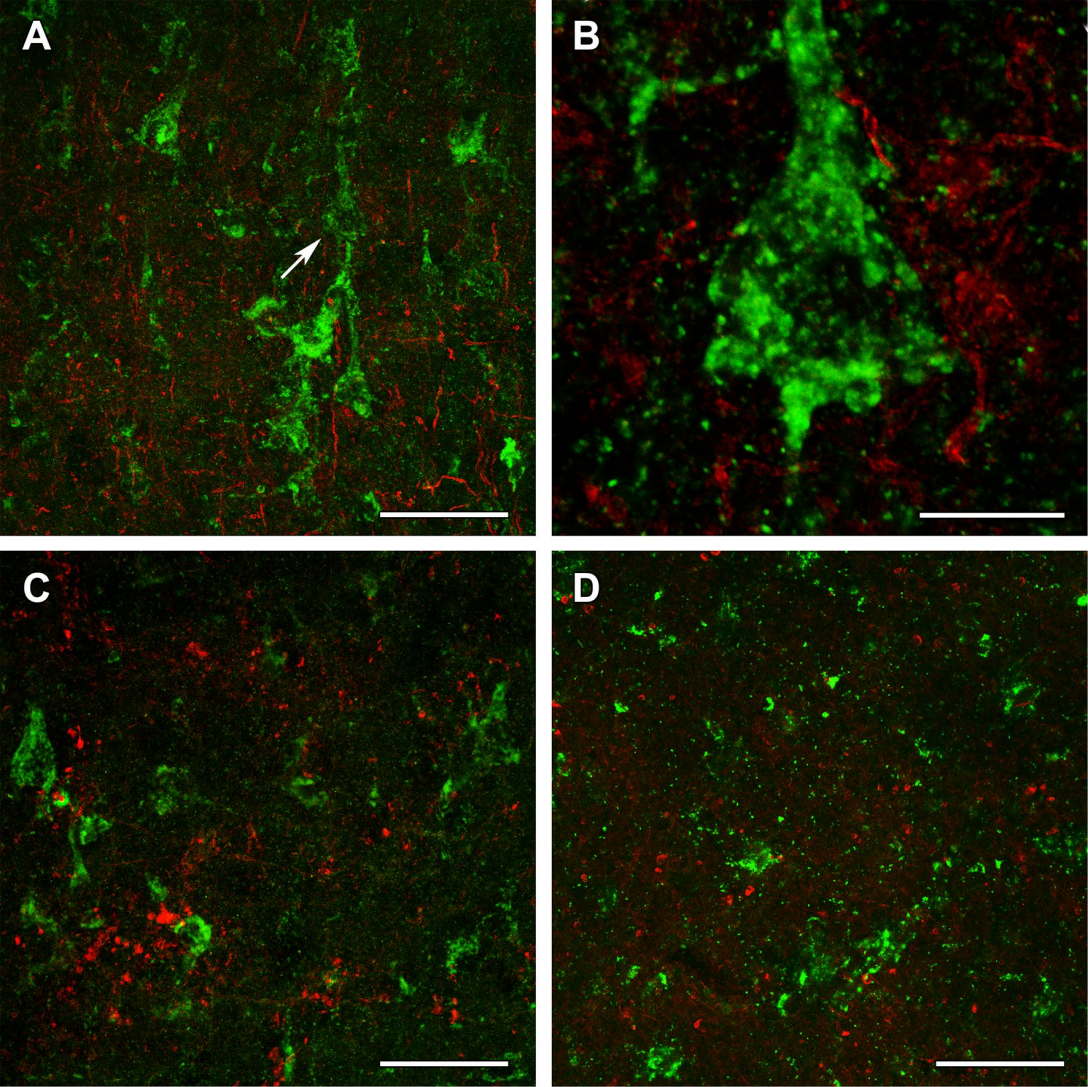
OGDHL and OGDH immunolabeling in the human cerebral cortex in relation to glial markers. **a** OGDHL-immunoreactive (green) cells as well as ALDHL1-positive (red) astrocytes are both present in the cerebral cortex without any visible co-localization. **b** A high magnification confocal image of the area pointed to by the white arrow in **a** demonstrates that OGDHL-ir cells are not labeled with Aldhl1 and in turn, Aldhl1-ir astrocytes do not contain OGDHL immunoreactivity. **c** Aldhl1 (red) is also absent in OGDH-ir cells and their processes (green). **d** The image demonstrates the lack of co-localization between OGDHL immunoreactivity (green) and the oligodendrocyte marker Olig2 (red). Scale bars = 50 μm for **a**, **c**, and **d**, and 10 μm for **b**

**Fig. 13:**
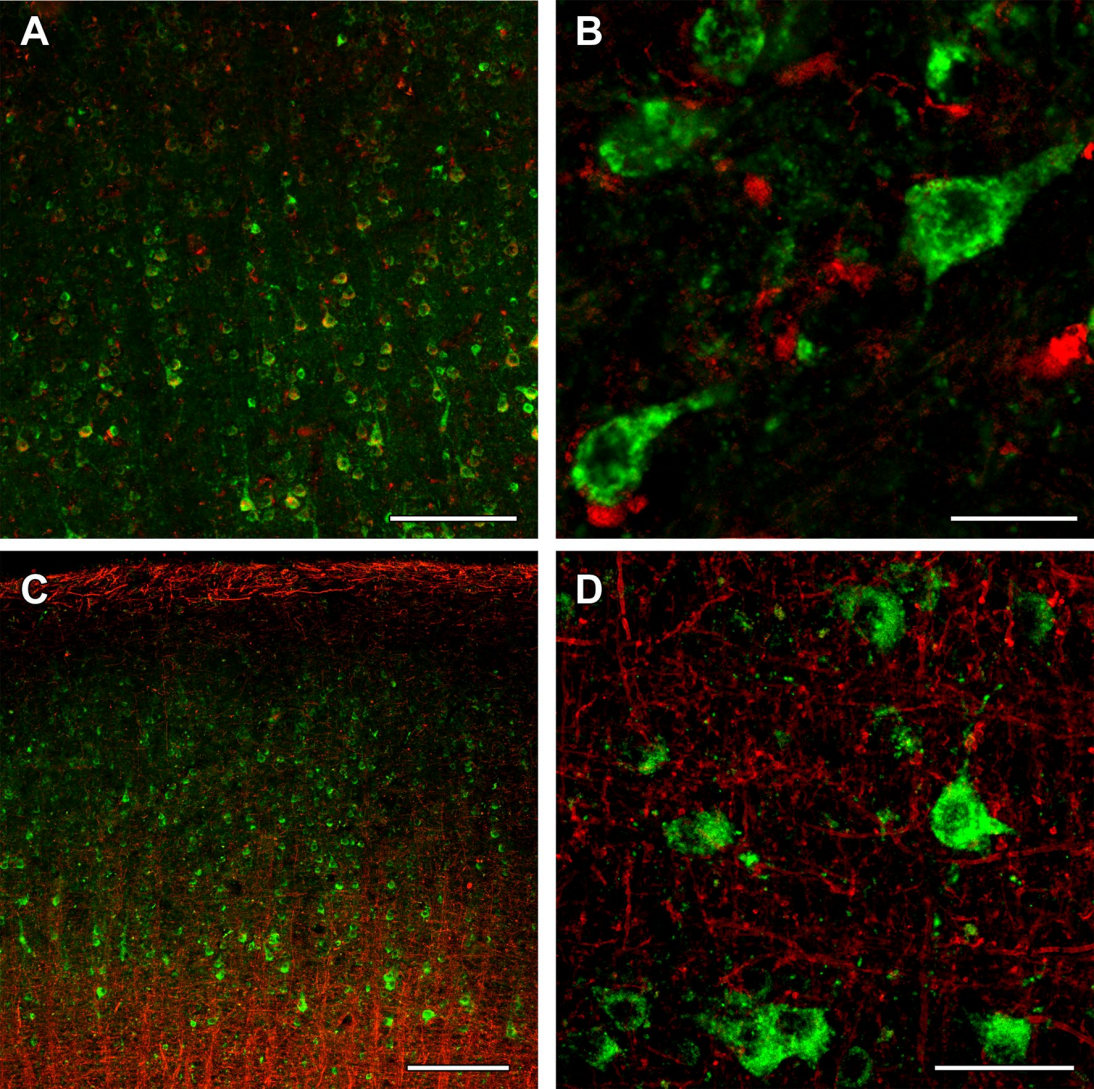
OGDH immunolabeling in the human cerebral cortex in relation to microglia and oligodendrocyte markers. **a** OGDH isoform 1/2 (green) and Iba1 (red) immunolabeling suggest a distinct distribution of OGDH-containing cells and microglia. In low magnification, OGDH- and IBA1-co-immunoreactivity may appear, but this notion is rejected in high magnification microphotographs. **b** A high magnification, optically 1 µm-thick confocal image of a cerebral cortical section double stained with OGDH isoform 1/2 (green) and Iba1 (red) demonstrates that larger, neuronal cells are single labeled for OGDH isoform 1/2. Several small cells are IBA1-positive. These cells are not labeled with OGDH isoform ½, suggesting that that microglial cells are negative for OGDH isoform 1/2. **c** A cerebral cortical section double labeled with OGDH isoform 1/2 (green) and the established oligodendrocyte marker myelin basic protein (MBP; red). The latter shows the typical distribution of myelinated fibers in the cerebral cortex. **d** A high magnification confocal image shows the lack of co-localization between OGDH isoform 1/2 and MBP-positive structures (red). Scale bars = 200 µm for **a** and **c**, 25 µm for **b**, and 50 µm for **d**

To identify the type of cells labeled with the different OGDH subtypes, double labeling was performed. A fluorescent Nissl dye allowed the analysis of cellular morphology, which suggested the presence of all OGDH subtypes in neurons. In addition, a high number of smaller cells, also labeled with fluorescent Nissl dye, were also present, but did not contain OGDH. Since the smaller size and the even distribution of these cells suggested that they may be glial cells, we performed double labeling with antibodies recognizing different OGDH subtypes and the astrocyte markers GFAP (Figs. 10 and 11) and Aldhl1 (Fig. 12). The results of these doubly labeling studies indicated the absence of OGDH immunoreactivity in astrocytes. Double labelling of OGDH subunits with the oligodendrocyte markers Olig2 (Fig. 12) and myelin basic protein (Fig. 13), or the microglial marker IBA1 (Fig. 13) also precluded localization of this KGDHC-specific subunit in oligodendroglia and microglia cells, respectively.

**Fig. 14:**
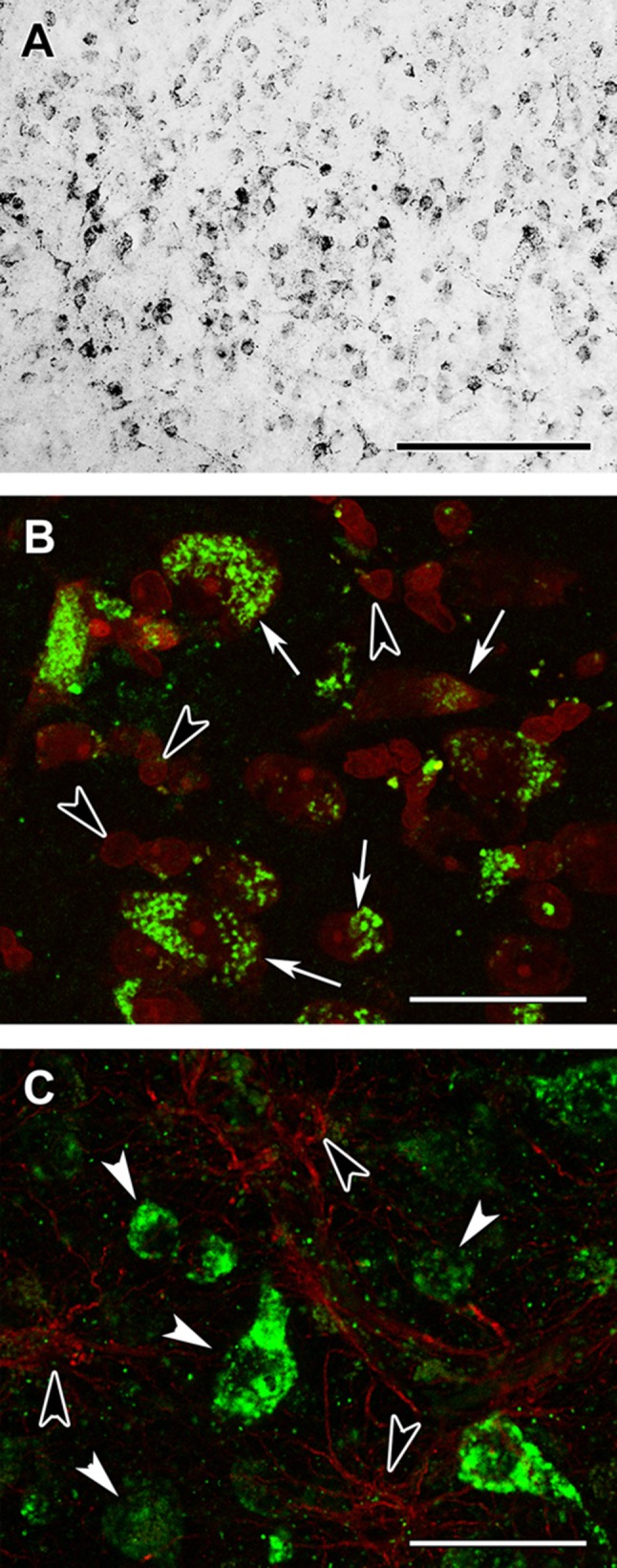
DLST immunolabeling in the human cerebral cortex in relation to neuronal and glial markers. **a** DLST-immunoreactive (DLST-ir) cells are present in the cerebral cortex, with higher density in the deep layers. **b** DLST (green) and fluorescent Nissl staining (red) show that many cells are double-labeled in the cerebral cortex. Arrows point to double-labeled neurons, while black arrowheads point to single-labeled, DLST-immunonegative cells. Please note the dot-like distribution of DLST immunolabeling in the double-labeled neurons. **c** A cerebral cortical section double labeled with DLST (green) and the established astrocyte marker glial fibrillary acidic protein (GFAP; red) to show the lack of double labeling, suggesting that astrocytes are devoid of DLST. White arrowheads point to single-labeled (DLST-ir) neurons, while black arrowheads point to single-labeled (GFAP-ir) astrocytes. Scale bars = 300 µm for **a**, and 30 µm for **b** and **c**

**Fig. 15:**
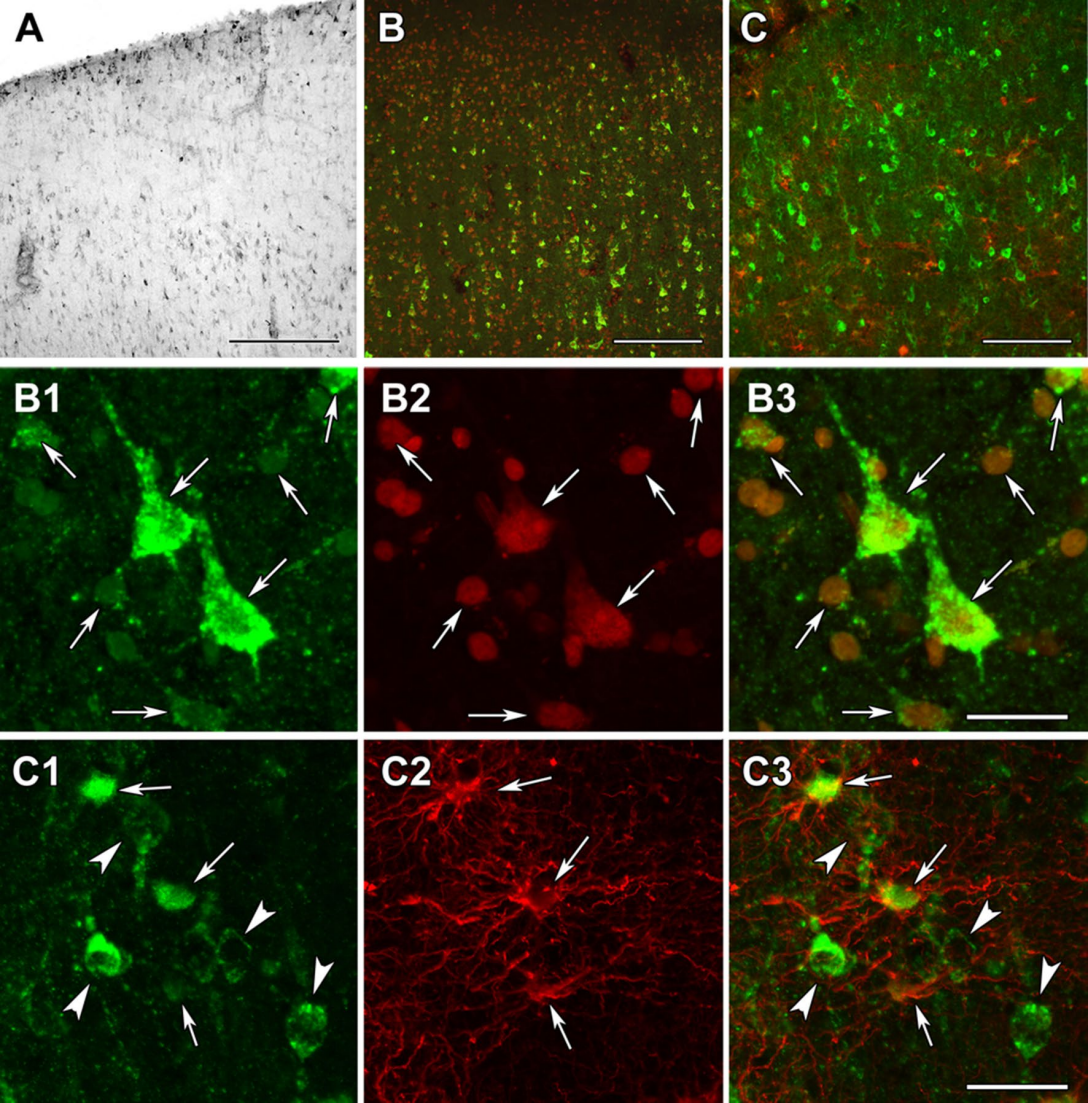
DLD immunolabeling in the human cerebral cortex in relation to neuronal and glial markers. **a** DLD-immunoreactive (DLD-ir) cells are present in layer 1 and also in deep layers of the cerebral cortex. The density of labeled cells is particularly high in layer 1 and the pyramidal layers. **b** DLD (green) and fluorescent Nissl staining (red) show that most cells are yellow, that is double-labeled in the cerebral cortex. **c** A cerebral cortical section double labeled with DLD (green) and the established astrocyte marker glial fibrillary acidic protein (GFAP; red) to show that most astrocyte contain DLD. **b**1–3 A high magnification confocal image of the cerebral cortical section double labeled with DLD (green) and fluorescent Nissl staining (red) demonstrates double labeling of the cells. The arrows point to some of the double-labeled cells. **b**1 shows the green channel, **b**2 the red channel, and **b**3 the merged figure. **c**1–3 A high magnification confocal image of the cerebral cortical section double labeled with DLD (green) and GFAP (red) demonstrates the presence of DLD in astrocytes (arrows). The arrowheads in turn point to neurons, which contain DLD, but do not express GFAP. **c**1 shows the green channel, **c**2 the red channel, and **c**3 the merged figure. Scale bars = 300 µm for **a**, 200 µm for **b**, 100 µm for **c**, and 20 µm for **b**1–3 and **c**1–3

Other subunits of KGDHC were examined in similar manner. DLST exhibited a distribution and localization resembling that of OGDH (Fig. 14a). In contrast, DLD was abundant in layer I as well as the deep layers of both the frontal and temporal cortices (Fig. 15a). Double labeling with the fluorescent Nissl dye suggested that all cells contain DLD (Fig. 15b). Indeed, astrocytes labeled with GFAP were shown to also localize DLD in them (Fig. 15c).

### Comparison of immunohistochemistry results obtained in this study from KGDHC subunit staining and those published in the Human Protein Atlas

The Tissue Atlas branch of the Human Protein Atlas aims to map the distribution of all human proteins in major tissues and organs, including the brain (Uhlen et al. 2015). The Tissue Atlas profiles proteins by antibodies in a qualitative manner. More specifically, antibodies are labeled with 3,3′-diaminobenzidine (DAB) yielding a brown discoloration of the labeled cell. Furthermore, the section is also counterstained with hematoxylin and cells are identified by visualizing their microscopical characteristics. With this approach, the spatial distribution, cell type specificity, and approximate relative abundance of proteins can be estimated, in the respective tissues. In Tables 4, 5, 6, 7, and 8, data from the Human Protein Atlas on brain tissues are shown when using KGDHC subunit- and isoform-specific antibodies. The Atlas categorizes staining as ‘high’, ‘medium’, or ‘low’, recognizing ‘glial’ and ‘neuronal’ staining. As shown in Tables 4, 5, 6, 7, and 8, in the human cortex, OGDH, OGDHL, and DLST are mostly expressed in neurons (medium or high staining), and they exhibit a low expression in glia. DLD staining is medium for both neurons and glia. It must be emphasized that visual identification of neurons versus glia by DAB followed by hematoxylin counterstain is prone to high false-positive and false-negative results. In the present work, cells were identified by Nissl, GFAP, IBA1, Olig2, Aldhl1, and myelin basic protein, affording a very high level of assurance regarding correct cell identification and categorization. Nevertheless, there is an obvious agreement between our data and those amassed by the Human Protein Atlas, namely that KGDHC-specific subunits are expressed in neurons and little (if at all) in cortical glia.

**Table 4:** Immunoreactivity of OGDH (using antibody HPA019514) in the human brain according to Human Protein Atlas

KGDH complex subunit	OGDH											
Antibody	Atlas Antibodies Cat#HPA019514, RRID:AB_1854772											
Isoform specificity	Q02218-1, Q02218-2, Q02218-3											
Brain region	Cerebral Cortex			Hippocampus			Caudate			Cerebellum		
Gender (M/F)	F	F	M	F	M	M	F	M	–	F	M	–
Age (years)	19	30	45	54	45	45	19	45	–	19	45	–
Glia (staining)	Low	Low	Low	Low	Low	Low	Low	Low	–	Low (molecular layer)	Low (molecular layer)	–
Neurons (staining)	High	High	High	Medium	Medium	Medium	Medium	Medium	–	Low granular layer, High Purkinje cells	Low granular layer, High Purkinje cells	–

**Table 5:** Immunoreactivity of OGDH (using antibody HPA020347) in the human brain according to Human Protein Atlas

KGDH complex subunit	OGDH											
Antibody	Atlas Antibodies Cat#HPA020347, RRID:AB_1854773											
Isoform specificity	Q02218-1, Q02218-2, Q02218-3											
Brain region	Cerebral Cortex			Hippocampus			Caudate			Cerebellum		
Gender (M/F)	F	F	M	F	M	M	M	M	M	F	F	M
Age (years)	19	30	57	54	45	45	45	45	70	19	54	45
Glia (staining)	Low	Low	Low	Medium	Medium	Medium	High	High	High	Medium (molecular layer)	Medium (molecular layer)	Medium (molecular layer)
Neurons (staining)	High	High	High	Medium	Medium	Medium	Medium	Medium	Medium	High (granular layer and Purkinje cells)	High (granular layer and Purkinje cells)	High (granular layer and Purkinje cells)

**Table 6:** Immunoreactivity of OGDHL in the human brain according to Human Protein Atlas

KGDH complex subunit	OGDHL											
Antibody	Atlas Antibodies Cat#HPA052497, RRID:AB_2681853											
Isoform specificity	Q9ULD0-1											
Brain region	Cerebral cortex			Hippocampus			Caudate			Cerebellum		
Gender (M/F)	M	M	M	F	F	F	M	M	M	F	F	M
Age (years)	6	37	70	42	52	64	37	58	70	19	54	54
Glia (staining)	Low	Low	Low	N.D.	N.D.	N.D.	N.D.	N.D.	N.D.	High (molecular layer)	High (molecular layer)	High (molecular layer)
Neurons (staining)	Medium	Medium	Medium	Low	Low	Low	Medium	Medium	Medium	High (Purkinje cells and granular layer)	High (Purkinje cells and granular layer)	High (Purkinje cells and granular layer)

*N.D.* not detected

**Table 7:** Immunoreactivity of DLST in the human brain according to Human Protein Atlas

KGDH complex subunit	DLST											
Antibody	Atlas Antibodies Cat#HPA003010, RRID:AB_1078679											
Isoform specificity	P36957-1, P36957-2											
Brain region	Cerebral Cortex			Hippocampus			Caudate			Cerebellum		
Gender (M/F)	F	F	M	F	F	M	F	F	M	F	M	–
Age (years)	45	54	45	19	54	45	19	54	45	19	45	–
Glia (staining)	Low	Low	Low	Medium	Medium	Medium	Medium	Medium	Medium	Medium (molecular layer)	Medium (molecular layer)	–
Neurons (staining)	Medium	Medium	Medium	Medium	Medium	Medium	N. INF.	N. INF.	N. INF.	Low in granular layer, high in Purkinje cells	Low in granular layer, high in Purkinje cells	–

*N.INF.* no information

**Table 8:** Immunoreactivity of DLD in the human brain according to Human Protein Atlas

KGDH complex subunit	DLD											
Antibody	Atlas Antibodies Cat#HPA044849, RRID:AB_2679111											
Isoform specificity	P09622-1, P09622-2, P09622-3											
Brain region	Cerebral cortex			Hippocampus			Caudate			Cerebellum		
Gender (M/F)	F	M	M	F	F	M	M	M	–	F	M	M
Age (years)	30	54	57	52	64	70	45	70	–	28	37	57
Glia (staining)	Medium	Medium	Medium	Medium	Medium	Medium	Medium	Medium	–	Medium (molecular layer)	Medium (molecular layer)	Medium (molecular layer)
Neurons (staining)	Medium	Medium	Medium	High	High	High	High	High	–	High (granular layer and Purkinje cells)	High (granular layer and Purkinje cells)	High (granular layer and Purkinje cells)

### Correlation of immunohistochemistry results obtained in this study from KGDHC subunit staining and RNA-Seq data published in the Allen Brain Atlas

**Fig. 16:**
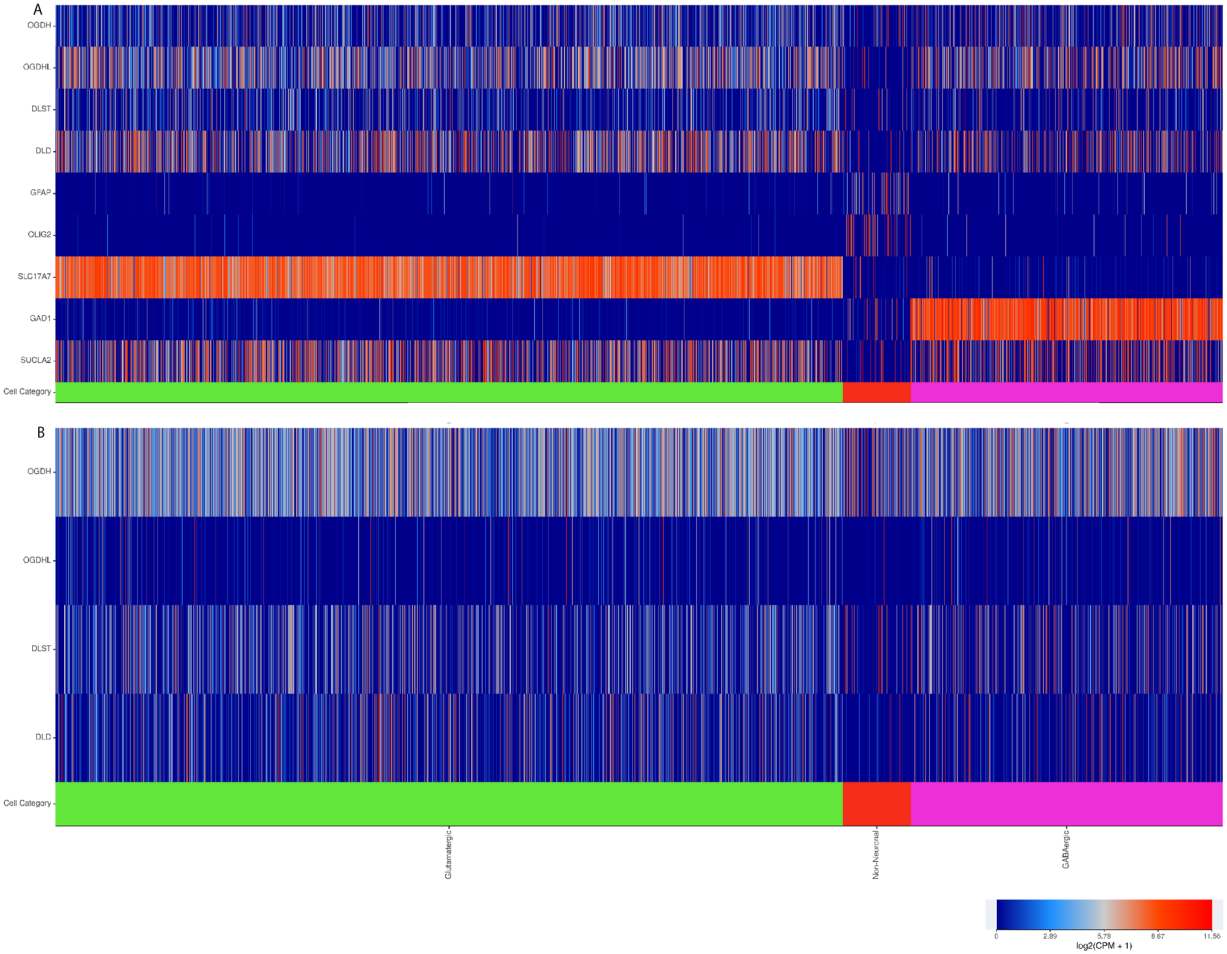
RNA-Seq data obtained from the Allen Brain Atlas, looking at the expression of genes indicated in the left, as a function of cell types. **a** Genes found only in exons. **b** Genes found only in introns. Image credit: Allen Institute. https://celltypes.brain-map.org/rnaseq/human

The Allen Brain Atlas is an integrated spatio-temporal portal for exploring the central nervous system (Sunkin et al. 2013). It entails—among other databases—RNA-Seq data from intact nuclei derived from frozen human brain specimens obtained from middle temporal gyri. Specifically, 15,928 nuclei from 8 human tissue donors ranging in age from 24–66 years were analyzed. As shown in Fig. 16a, RNA-Seq data for OGDH, OGDHL, DLST, and DLD (indicated on the left) are shown as a function of cell subtype (indicated in the *x*-axis). These data are pooled exclusively from the exons. It is immediately apparent that, in non-neuronal cells, thus including glia- expression of KGDHC components is much less than that in glutamatergic + GABA-ergic cells, i.e., collectively neuronal cells. For comparisons, RNA-Seq data for GFAP and Olig2 (glial markers), SLC17A7 (Solute Carrier Family 1 (Glutamate Transporter), Member 7; it transports L-glutamate; glutamatergic neuron marker), GAD1: Glutamate Decarboxylase isoform 1; GABA-ergic neuronal marker and SUCLA2 (component of ATP-forming succinate-CoA ligase, neuronal marker) are also presented. On the other hand, in Fig. 16b, RNA-Seq data from introns only are presented, and there, it is apparent that gene elements coding for KGDHC components are essentially the same between neuronal vs non-neuronal cells; this practically means that in non-neuronal cells (such as glia), genes coding for KGDHC components are embedded in intronic regions as a result of some genetic regulation.

### Protein lysine succinylation is pancellularly evident in the human brain

**Fig. 17:**
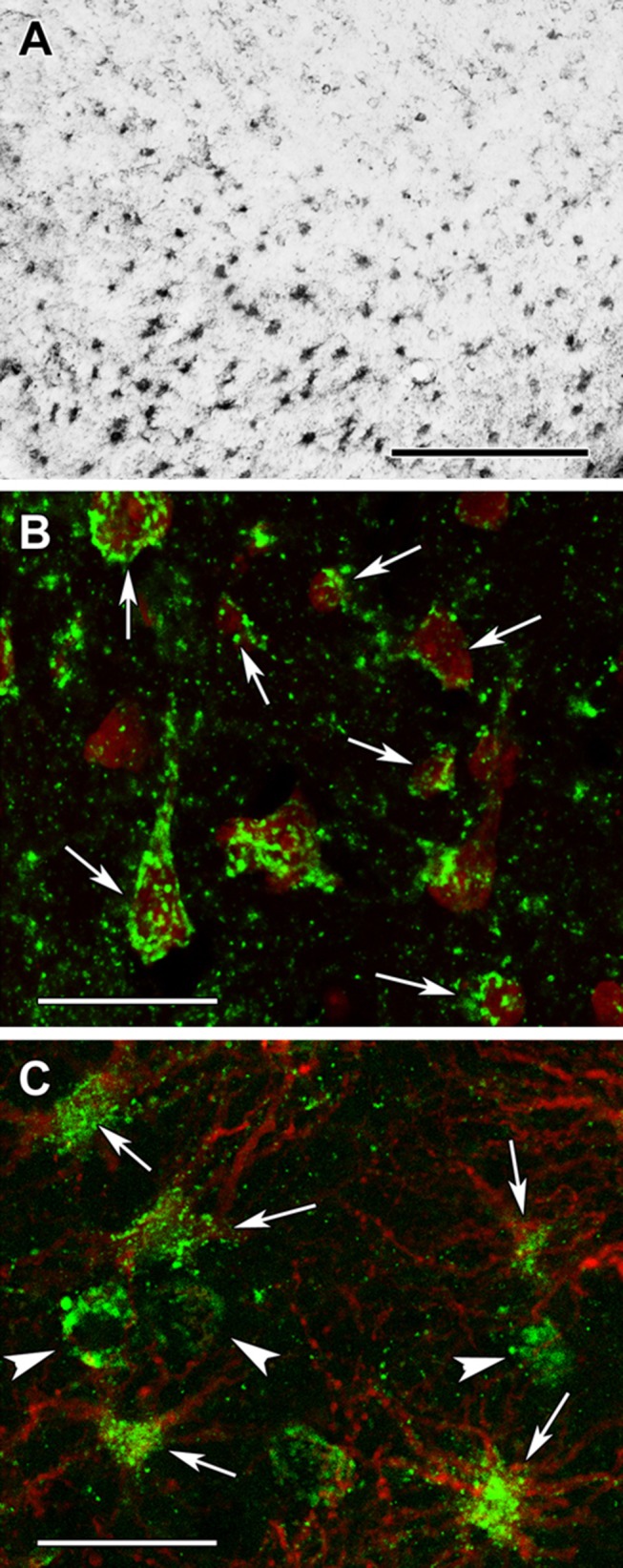
Succinyl-lysine immunolabeling in the human cerebral cortex in relation to neuronal and glial markers. **a** Succinyl-lysine-immunoreactive (SUCCLYS-ir) cells are present in the cerebral cortex, with higher density in the deep layers. **b** Succinyl-lysine (green) and fluorescent Nissl staining (red) show that essentially all cells are double-labeled in the cerebral cortex. Arrows point to some of these double-labeled cells. Note the dot-like distribution of succinyl-lysine immunolabeling in the double-labeled cells. **c** A cerebral cortical section double labeled with succinyl-lysine (green) and the established astrocyte marker glial fibrillary acidic protein (GFAP; red) to show the double labeling of astrocytes. White arrowheads point to single-labeled (SUCCLYS-ir) neurons. Scale bars = 300 µm for **a** and 30 µm for **b** and **c**

**Fig. 18:**
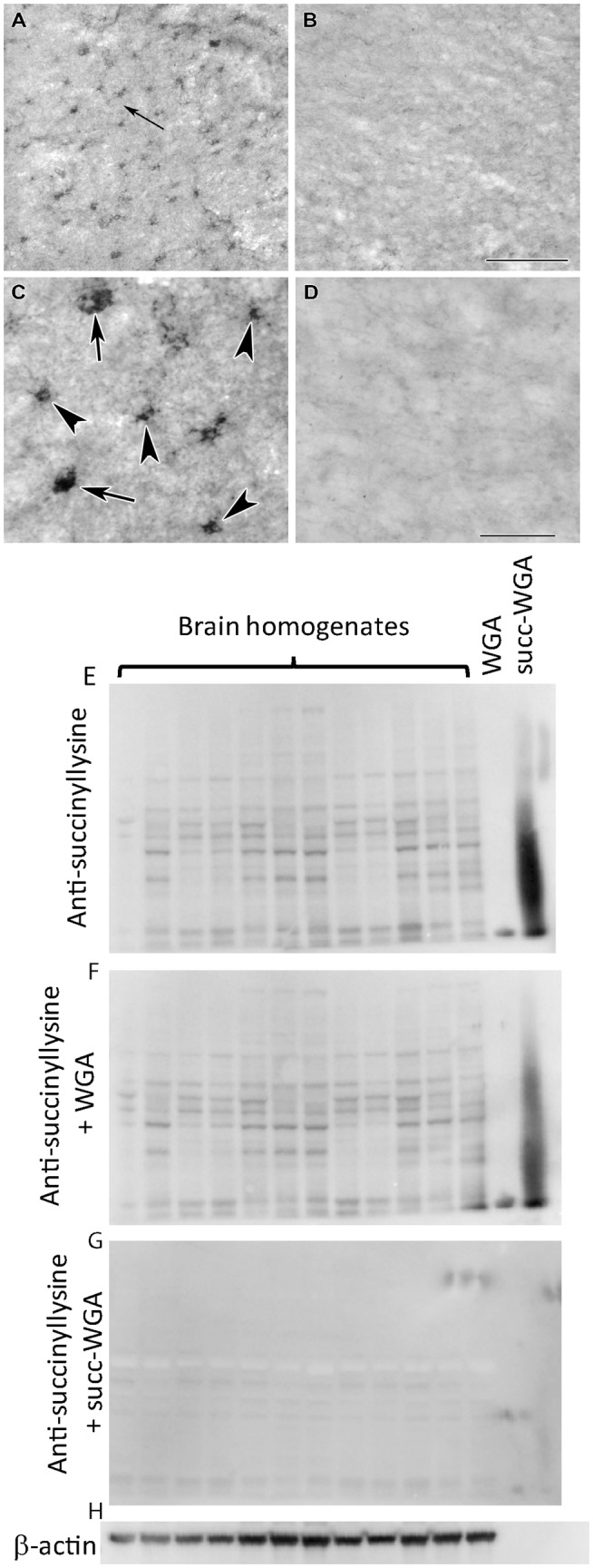
Testing of the specificity of the anti-succinyl-lysine antibody by absorption in the human cerebral cortex. **a** Preincubation of the anti-succinyl-lysine antibody with wheat germ agglutinin does not prevent immunolabeling. The arrow indicates the area magnified in **c**. **b** Following preincubation of the anti-succinyl-lysine antibody with succinylated wheat germ agglutinin, no labeled cells are present suggesting that the preincubation eliminates specific immunolabeling in a section adjacent to the one shown in **a**. **c**, **d** Higher magnification images confirm the effect of preabsorption with succinylated wheat germ agglutinin. Note the different types of labelled cells in **c** as arrows point to larger cells, probably neurons while arrowheads point to small cells with several processes resembling glial cells. Scale bars = 200 μm for **a** and **b**, and 50 μm for **c** and **d**. **e** Western blot loaded with 12 brain homogenates, WGA-loaded lane and succinylated WGA, where indicated, treated with anti-succinyllysine. **f** Same as for **e**, treated with WGA plus anti-succinyllysine. **g** Same as for **e** but treated with succinylated WGA plus anti-succinyllysine, which effectively eliminated the labeling. **h** β-actin as loading control

All cells in the examined cerebral cortices were immune-positive for protein lysine succinylation (Fig. 17a) as demonstrated by the lack of cells singly labelled with the fluorescent Nissl dye (Fig. 17b). The localization in astrocytes was also confirmed by double labeling with GFAP as indeed all GFAP-positive glial cells contained immunolabeling for protein lysine succinylation, as well (Fig. 17c). The specificity of the labeling was confirmed by the absence of signal when the antibody was pre-incubated with succinylated Wheat Germ Agglutinin (WGA, Fig. 18b, d) but not WGA without succinylation Fig. 18a, c). Specificity of the anti-succinyllysine antibody was further assured by an independent methodology, Western blotting (WB) of homogenized brains. As shown in Fig. 18e–h, WB for 12 different brain homogenates was performed. In the same gels, wheat germ agglutinin (WGA) and succinylated WGA were loaded where indicated. In panel E, blots were probed with anti-succinyllysine antibody; the blot shown in panel F was treated with anti-succinyllysine antibody plus WGA; the blot shown in panel G was treated with anti-succinyllysine antibody plus succinylated WGA; and the blot strip shown in panel H depicts the result of probing for β -actin, as loading control. It is evident that incubation of the anti-succinyllysine antibody with succinylated WGA (Fig. 18g) but not WGA (Fig. 18f) yields very little staining. WGA contains endogenous succinylated sites (Zhang et al. 2017), and thus, a band is visible in the respective lane.

### Proposed metabolic differences (pertinent to KGDHC) between neurons and glia in the adult human cortex

**Fig. 19:**
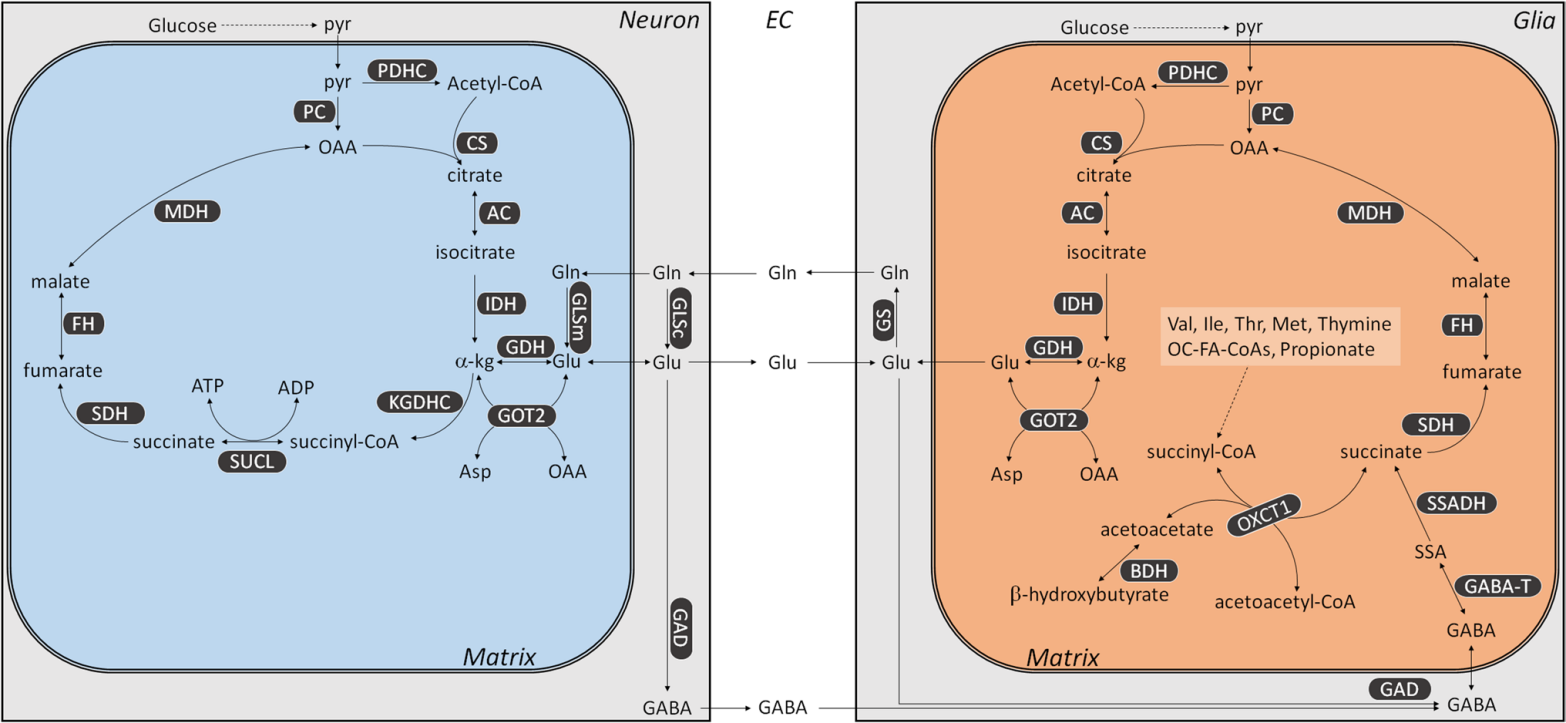
Metabolic considerations of a neuronal mitochondrion (blue box) and a glial mitochondrion (orange box) pertinent to KGDHC. *AC* aconitase, *BDH* β-hydroxybutyrate dehydrogenase, *CS* citrate synthase, *FH* fumarase, *GAD* glutamate decarboxylase, *GABA-T* GABA aminotransferase, *GDH* glutamate dehydrogenase, *GLSc* glutaminase (cytosolic), *GLSm* glutaminase (mitochondrial), *GOT2* aspartate aminotransferase, *IDH* isocitrate dehydrogenase, *KGDHC* ketoglutarate dehydrogenase complex, *MDH* malate dehydrogenase (mitochondrial), *OXCT1* succinyl-CoA:3-oxoacid CoA-transferase, *PC* pyruvate carboxylase, *PDHC* pyruvate dehydrogenase complex, *SDH* succinate dehydrogenase, *SSADH* succinic semialdehyde dehydrogenase, *SUCL* succinyl-CoA ligase

The above results indicate that glia of the human brain lack KGDHC. Mindful of the previously published work shows that they also lack succinate-CoA ligase (Dobolyi et al. 2015a; b), and the segment of the citric acid cycle from α-ketoglutarate to succinate could only be connected through the GABA shunt, a pathway which is known to take place in glia (Minelli et al. 1996; Conti et al. 1998, 1999). However, succinyl-CoA is still being made since protein lysine succinylations were detected in these cells as well. Thus, we propose the following metabolism pertinent to KGDHC in neurons versus glia: as shown in Fig. 19, neuronal mitochondria (blue box) perform the citric acid cycle as described in textbooks. They also receive glutamine (Gln) from the extracellular space (EC), which originated from glia as part of the so-called “glutamate–glutamine cycle” between neurons and glia (Sonnewald and Schousboe 2016). Some neurons will also extrude GABA to the EC, which can be taken up by the glia. Glial mitochondria (orange box), on the other hand, will also take up glutamate from the EC, which has been released by neurons and further process it by transamination or use it for forming GABA. Succinyl-CoA in glia can only be produced from valine, isoleucine, methionine, thymine, odd-number chain fatty acids, and, perhaps, propionate (multiple reactions, omitted for clarity), and from the reaction catalyzed by OXCT1, an enzyme participating in ketone bodies catabolism; relevant to this, ketone bodies metabolism is known to occur in human astrocytes (Thevenet et al. 2016).

## Discussion

The two most important observations of the present work are that (1) we detected KGDHC-specific subunits immunoreactivity in neurons but not glia of the human brain, and (2) protein lysine succinylations were pancellularly observed, thus, in glia, succinyl-CoA is probably a product of an enzyme other than KGDHC. Furthermore, to the best of our knowledge, our work is the first to demonstrate protein lysine succinylation in the adult human cortex. DLD subunit was found also in non-neuronal cells, but this is probably due to its participation in the pyruvate dehydrogenase complex, branched-chain alpha-keto acid dehydrogenase complex, and the glycine cleavage system.

Regarding the finding that KGDHC-specific components were not observed in cells staining positive with glial markers, it is important to emphasize that our immunohistochemistry protocols included several amplification steps. This implies that if KGDHC-specific components are expressed in human glia, they are probably at least two orders of magnitude less abundant than those expressed in neurons, precluding the possibility of an appreciable KGDHC activity in the former cell types. The group of John P Blass has also published the absence of KGDHC immunoreactivity from glia in the adult human brain; however, out of perhaps abundance of caution, they reported it as “surprisingly, convincing immunoreactivity was not found in glia” (Ko et al. 2001), even though KGDHC immunoreactivity was completely absent from all of their microphotographs. Here, we investigated glial cells exhibiting GFAP, Aldhl1, myelin basic protein, Olig2, and IBA1, identifying astrocytes, oligodendrocytes, and microglia. However, the human brain harbors hundreds (if not thousands) of cell types and subtypes. The Human Brain Project aims to identify and catalogue all these cells (Amunts et al. 2016). Thus, it cannot be excluded that some glial subtypes exhibit KGDHC-specific components to a detectable level. This is supported by RNA-Seq data obtained from the Allen Brain Atlas, in which positive “hits” were scarce but still visible in non-neuronal cells. The notion that glia of the adult human brain may not exhibit KGDHC (nor succinyl-CoA ligase) activity, thus precluding operation of the citric acid cycle as we know it calling for reconsideration of a textbook definition of a universal metabolic pathway should not come as a great surprise: mice engineered to lack cytochrome c oxidase in astrocytic mitochondria in vivo were fully viable in the absence of any signs of glial or neuronal loss even at 1 year of age (Supplie et al. 2017). Notably though, KGDHC (detected immunologically using an antibody generated using the whole complex as an epitope) has been reported in astrocytes of cerebrum and cerebellum of newborn mice (Waagepetersen et al. 2006). Furthermore, in (Calingasan et al. 1994), it was shown that in 3-month-old male Fischer 344/Brown Norway F1 hybrid rats immunohistochemistry for KGDHC (using an anti-rabbit IgG for KGDHC, no further information is provided), KGDHC stained neurons much more intensely than glia in several brain areas. Perhaps, KGDHC is absent (or at least present at an extremely small level) in human glia so as to assist the glutamate-glutamine cycle by preventing glutamate catabolism through the citric acid cycle (which necessitates KGDHC activity), instead being shuttled towards glutamine formation. By the same token, glutamate taken up from the EC would be shuttled towards GABA metabolism, also known to operate in glia cells. Relevant to this, in a human embryonic kidney cell line, even a mild reduction (~ 30%) in KGDHC activity elevated the GABA shunt (Shi et al. 2009).

Regarding the observation of pancellular lysine succinylation in the adult human cortex in view of the fact that glia may not exhibit KGDHC nor succinyl-CoA ligase activity implies that these cells must obtain succinyl-CoA from some other metabolic pathway. Succinylations are widespread and may occur in an enzymatic (Gibson et al. 2015; Wang et al. 2017; Kurmi et al. 2018) and non-enzymatic (Wagner and Payne 2013; Weinert et al. 2013) manner. However, they all require succinyl-CoA. In mammals, this high-energy metabolite can be generated only through KGDHC, succinyl-CoA ligase, catabolism of valine, isoleucine, methionine, thymine, odd-number chain fatty acids (and perhaps propionate), and through the reaction catalyzed by OXCT1. Since glia may not exhibit KGDHC nor succinyl-CoA ligase components, provision of succinyl-CoA is ensured only by the remaining pathways. Mindful of the remaining pathways generating succinyl-CoA inside the matrix, it is still a mystery how can there be extramitochondrial protein lysine succinylations. KGDHC components have been observed in the nuclei of the human cell line U251 (Wang et al. 2017), in rat neonatal cardiac myocytes, human-induced pluripotent stem cardiac myocytes, SW620 human colon cancer cells, and developing mouse embryonic heart (Choi et al. 2019); however, we did not observe KGDHC subunit immunoreactivity in the nuclei of our human fibroblasts, HeLa, and COS-7 cells, nor in the human cortical sections.

## Electronic supplementary material

Below is the link to the electronic supplementary material.

Supplementary file1 (PDF 11323 kb)

Supplementary file2 (DOCX 12 kb)
