# Supplementary material for: Exclusive neuronal detection of KGDHC-specific subunits in the adult human brain cortex despite pancellular protein lysine succinylation
Source: Brain Struct Funct. 2020 Jan 25;225(2):639–67. doi: 10.1007/s00429-020-02026-5 (PMC7046601; doi:10.1007/s00429-020-02026-5)
Supplement: Supplementary file 1 — Supplementary file1 (PDF 11323 kb) [file 429_2020_2026_MOESM1_ESM.pdf]

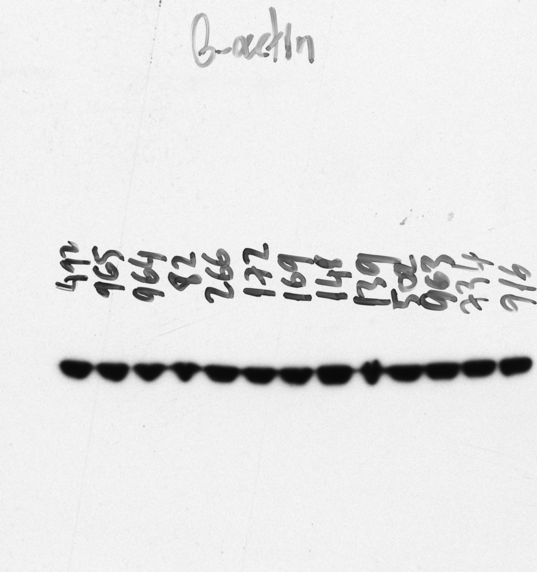
β-actin

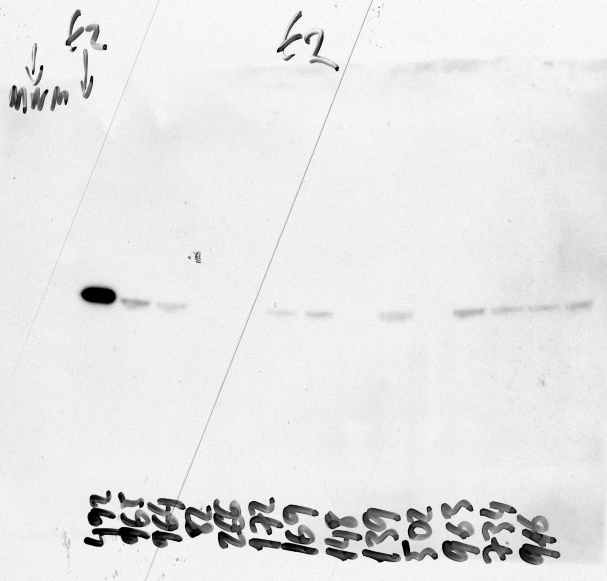

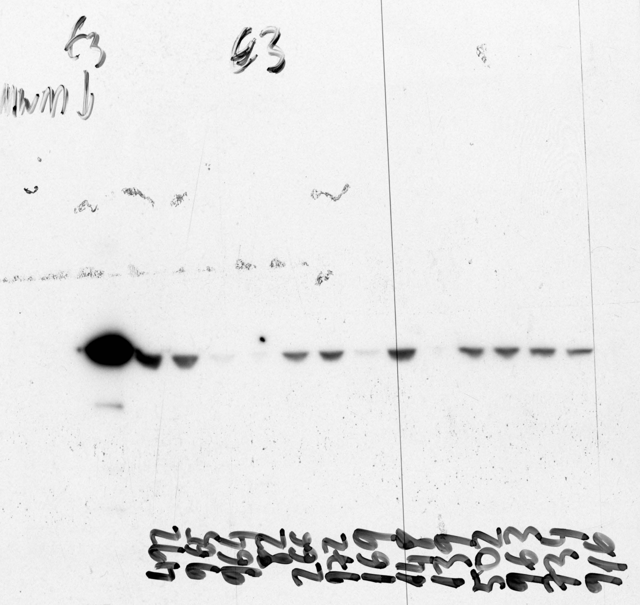

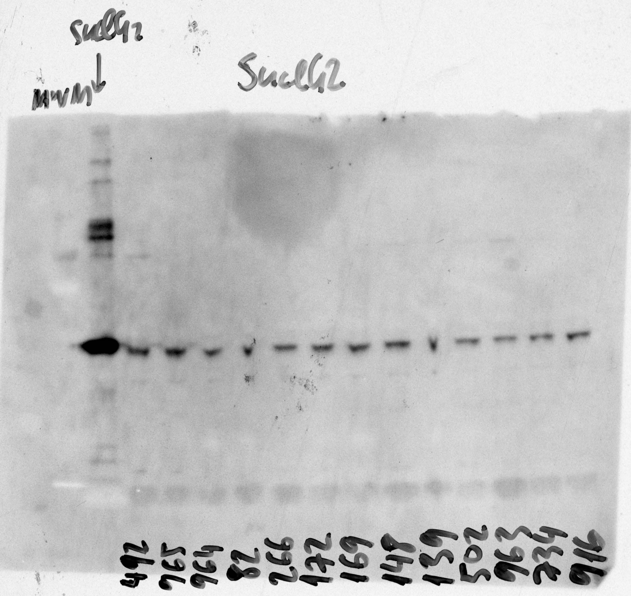
SuclG2
MWM
SuclG2

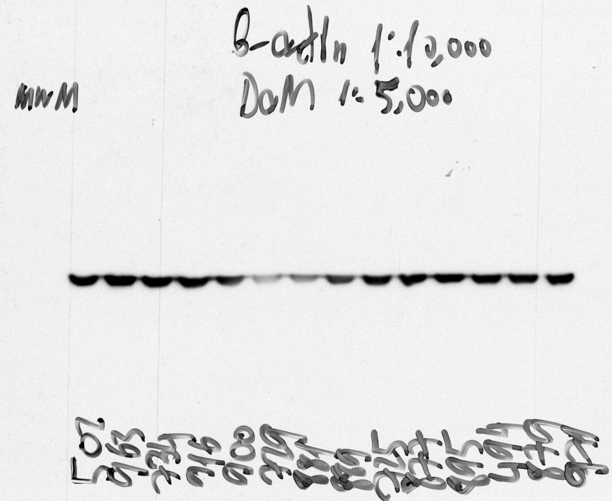
β-actin 1:10,000
DαM 1:5,000
MWM

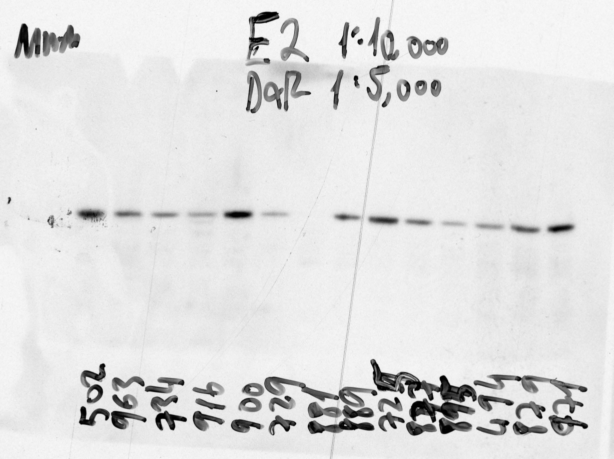
E2 1:10,000
DaR 1:5,000

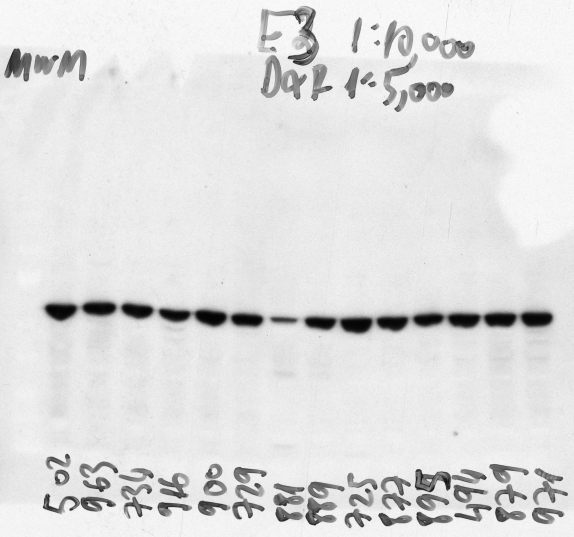
MWM
E3 1:10,000
DαR 1:5,000

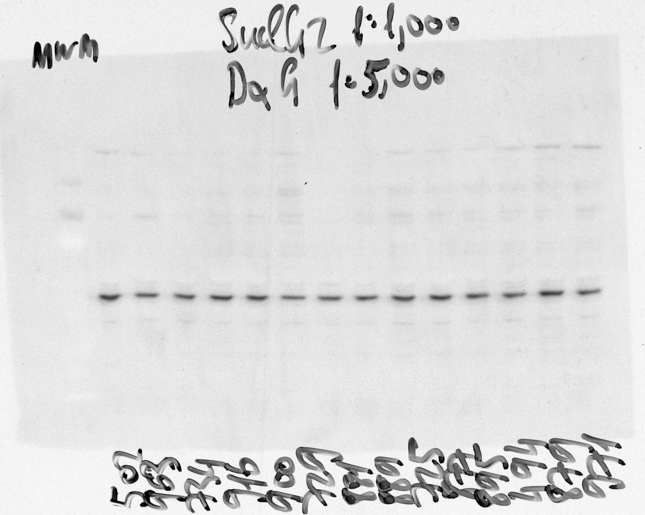
MWM
SuelG2 1:1,000
Dα G 1:5,000

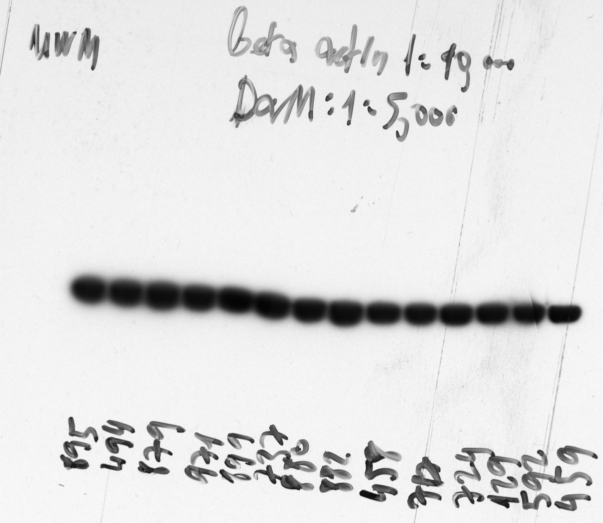
MWM
Beta actin 1:10,000
DaM: 1:5,000

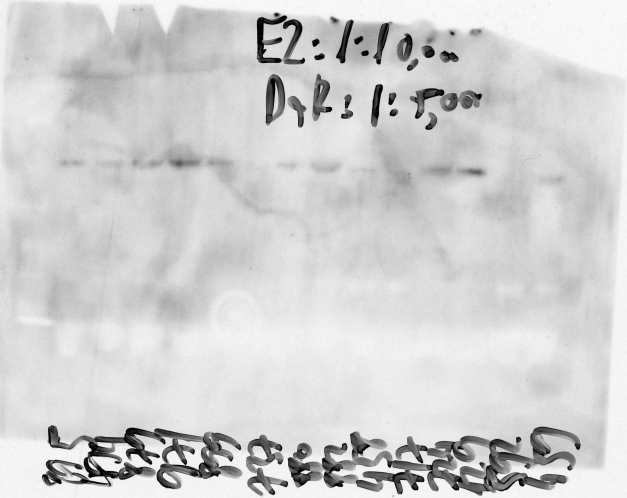
E2: 1:10,000
DαR: 1:5,000

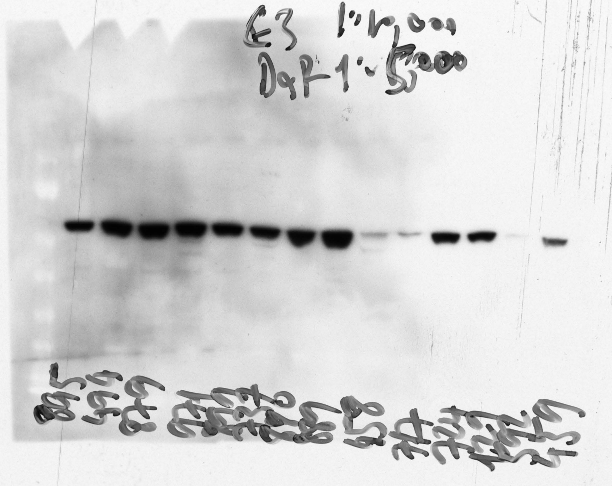
E3 1:1,000
DαR 1:5000

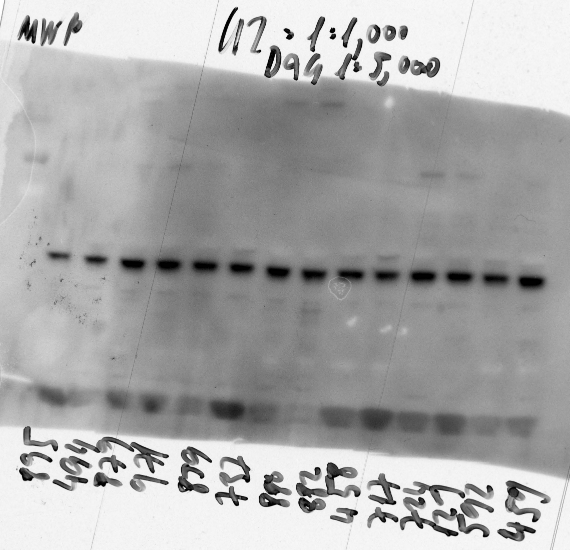
MWP
U2 : 1:1,000
D9G 1:5,000

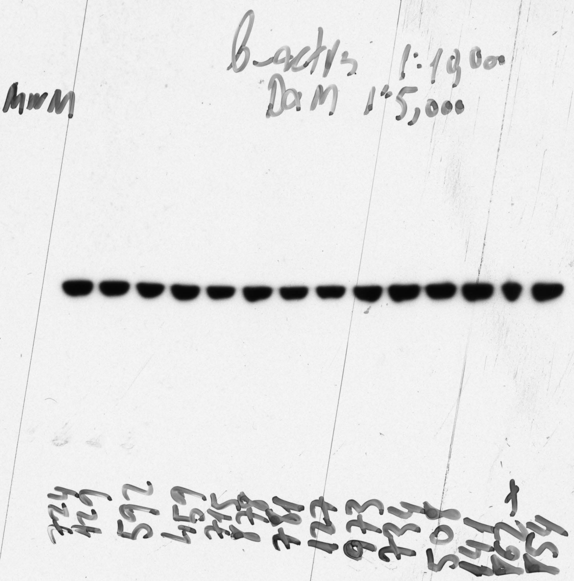
β-actin 1:10,000
DaM 1:5,000
MWM

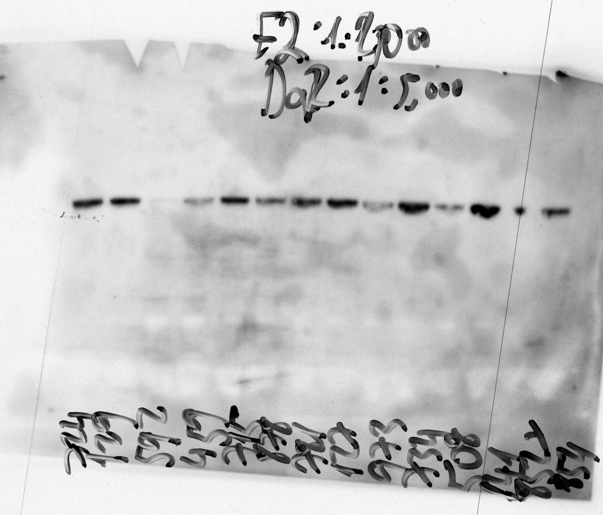
F2 : 1:2000
DaR : 1 : 5,000

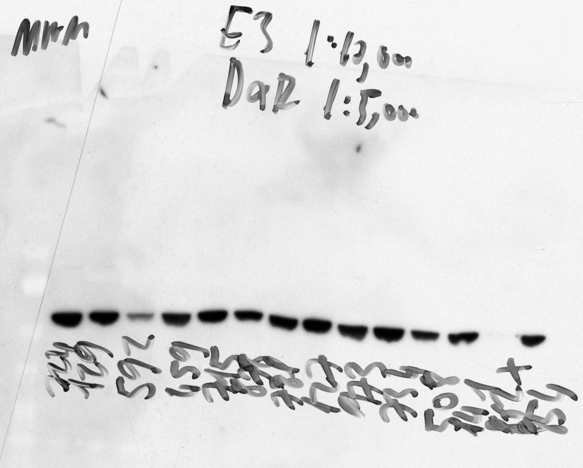
E3 1:10,000
DaR 1:5,000

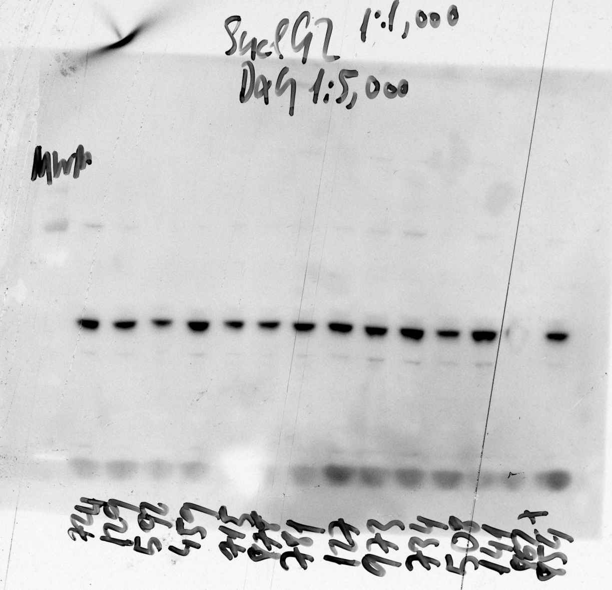
SuclG2 1:1,000
DaG 1:5,000
MWM

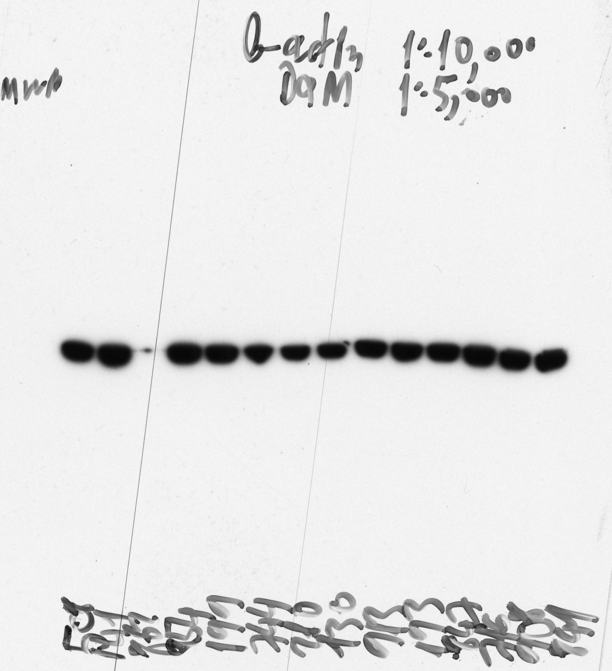
MWM
b-actin 1:10,000
DαM 1:5,000

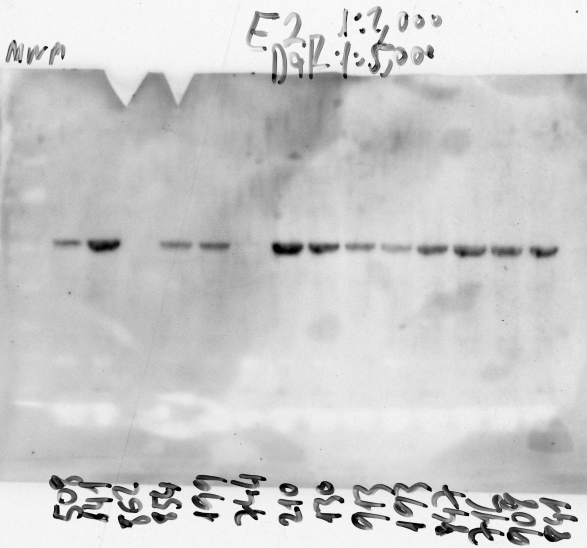
MWM
E2 1:2,000
DαR 1:5,000

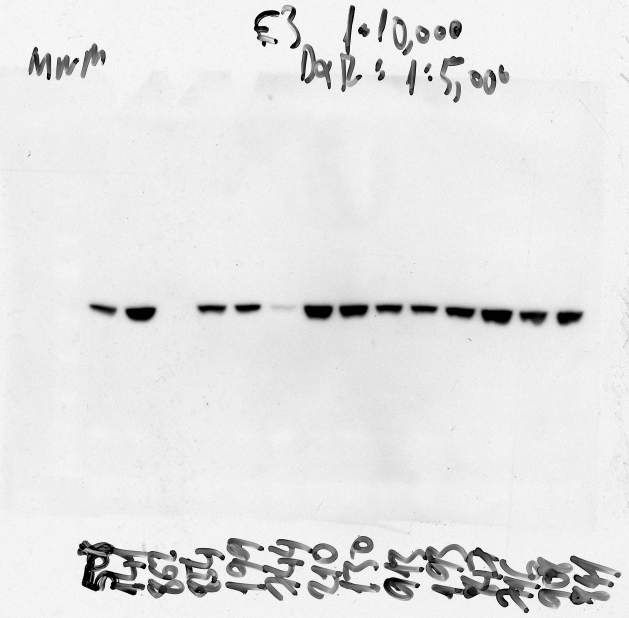
MWM
E3 1:10,000
DαR : 1:5,000

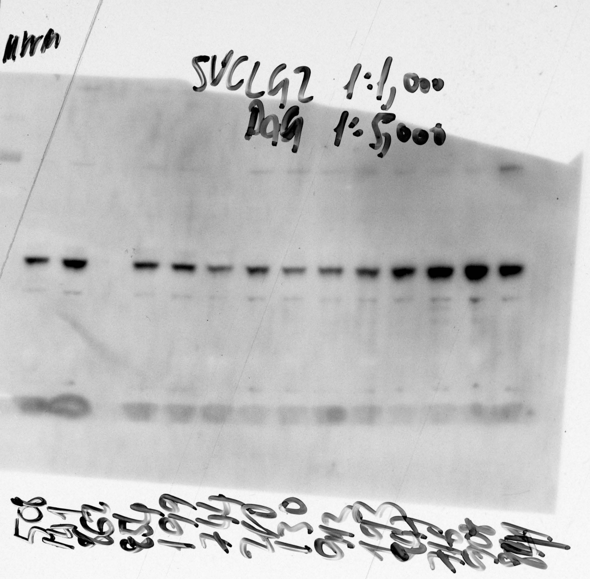
MWM
SVCLG2 1:1,000
DaG 1:5,000

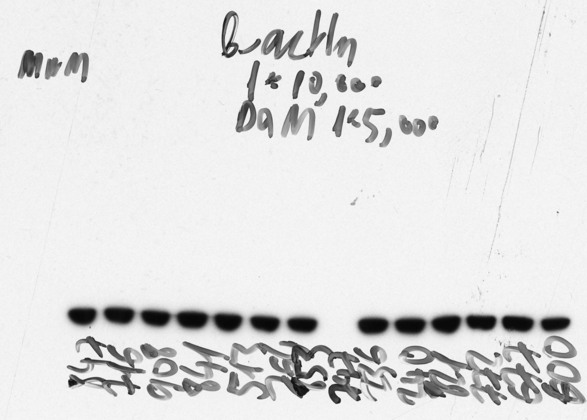
MnM
B-actin
1:10,000
DaM 1:5,000

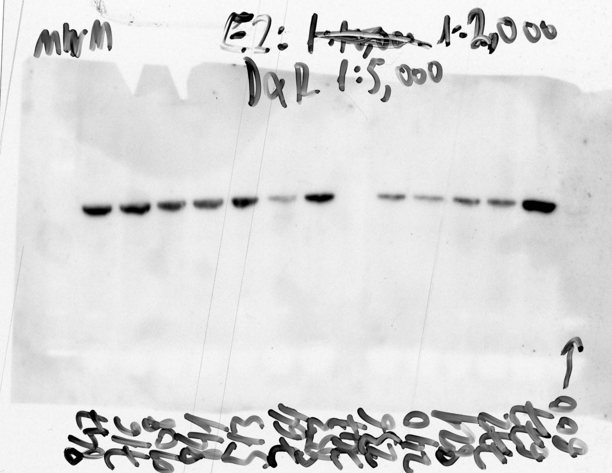
MWM
E2: 1:2,000
DαR 1:5,000

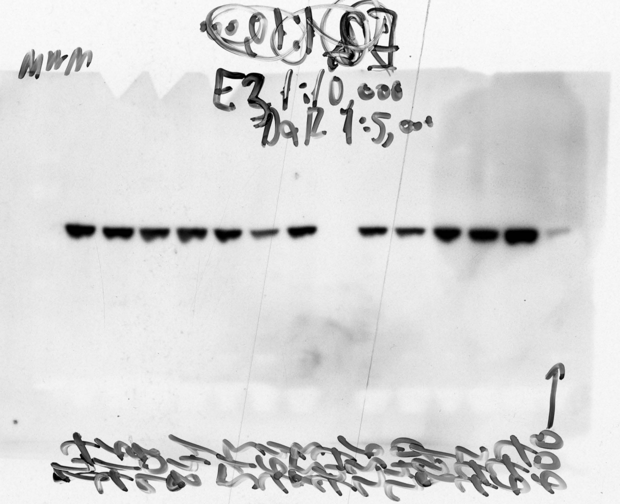
MWM
E3 1:10,000
DaR 1:5,000

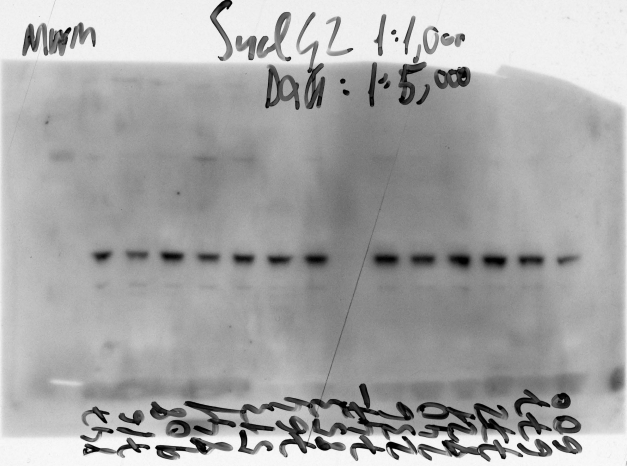
MWM
SucLG2 1:1,000
DαG : 1:5,000

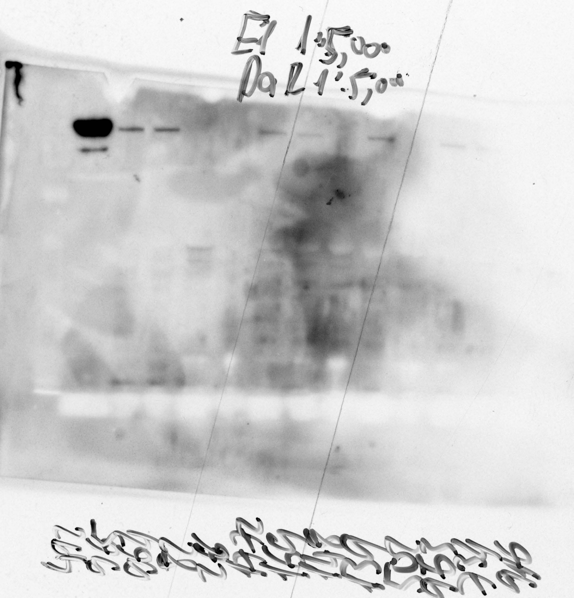
E1 1:5,000
DaR 1:5,000

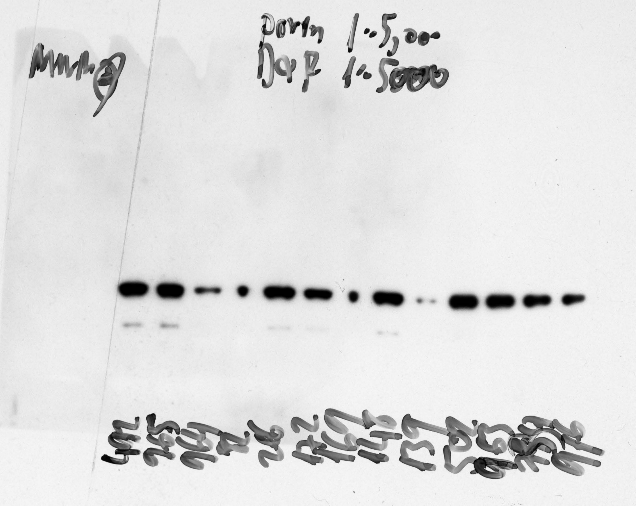
1:5,000
1:5000

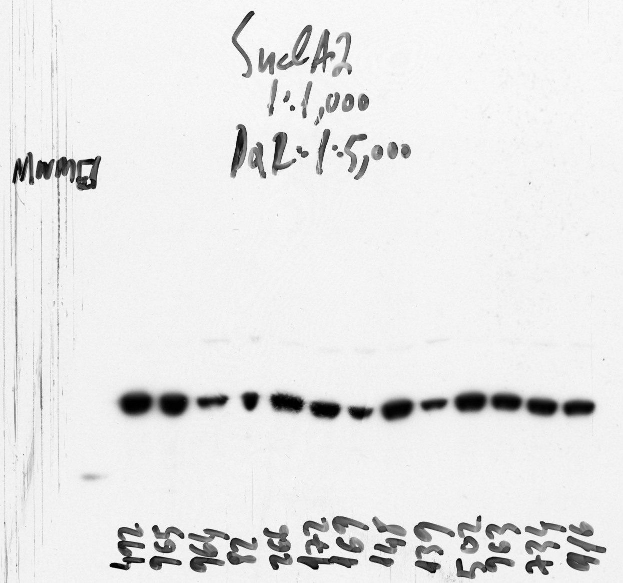

SuclA2
1:1,000
DaR: 1:5,000

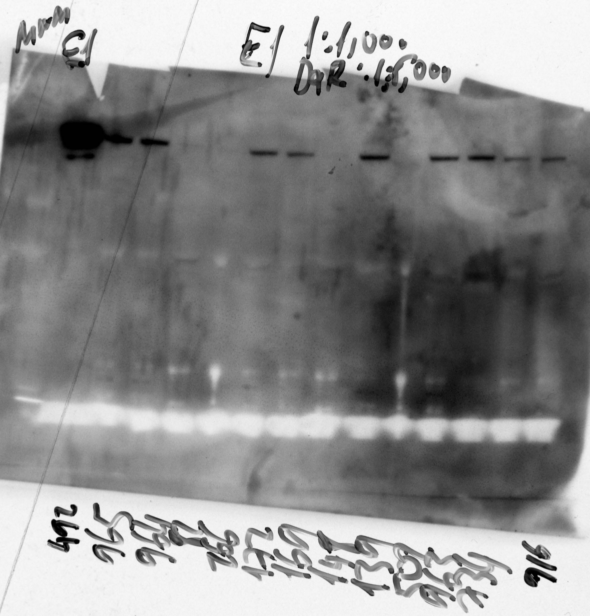
C1
E1 1:1,000
DAR 1:5,000

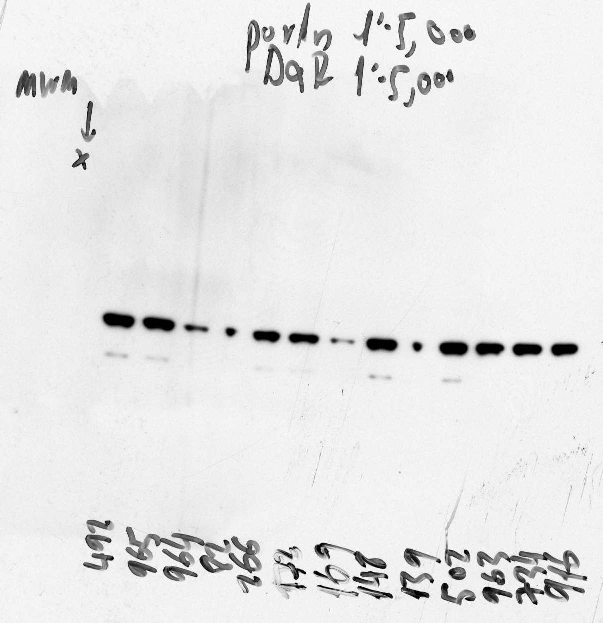
porin 1:5,000
DαR 1:5,000
MWM

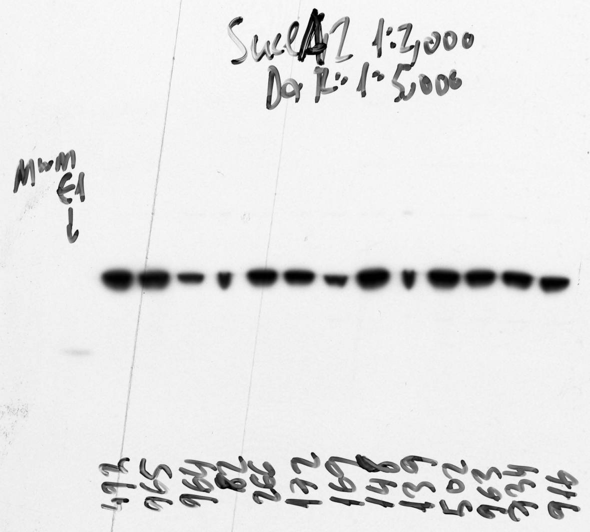
SucA2 1:2,000
Da R: 1:5,000
MWM
E1

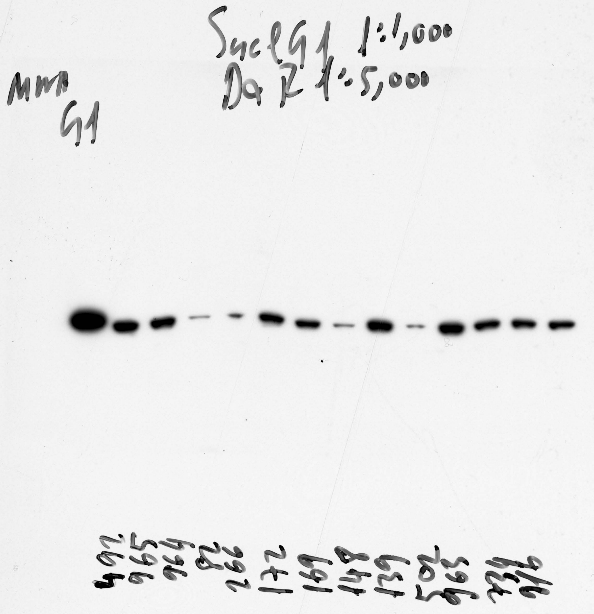
SuclG1 1:1,000
Da R 1:5,000
G1

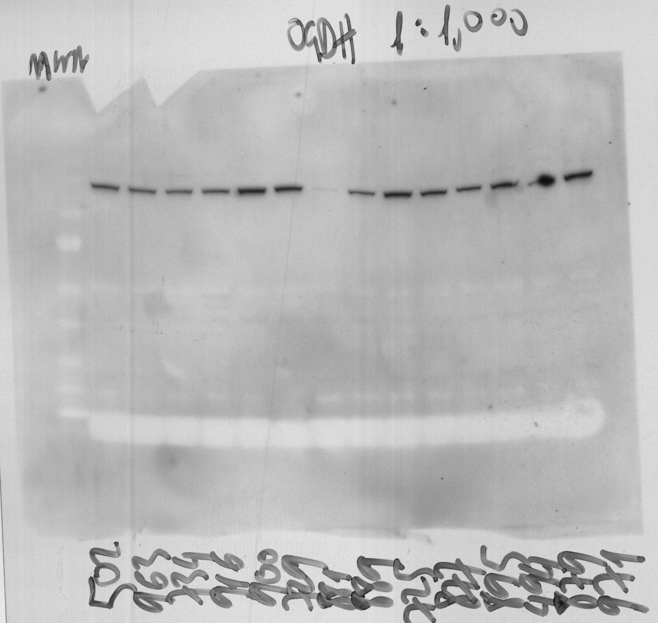
OGDH 1:1,000
MWM

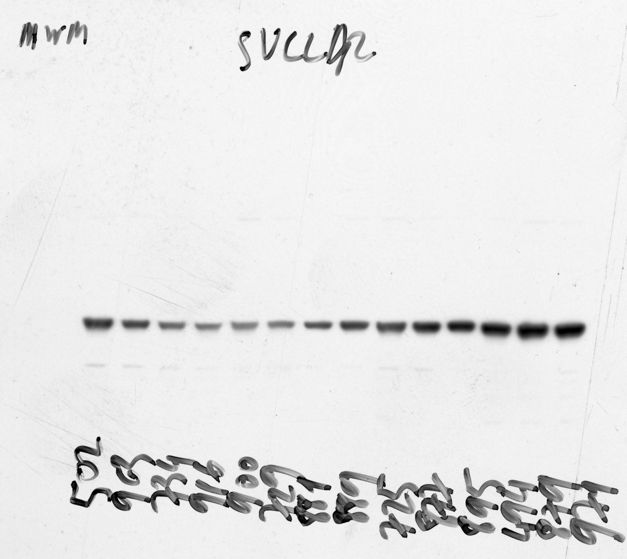

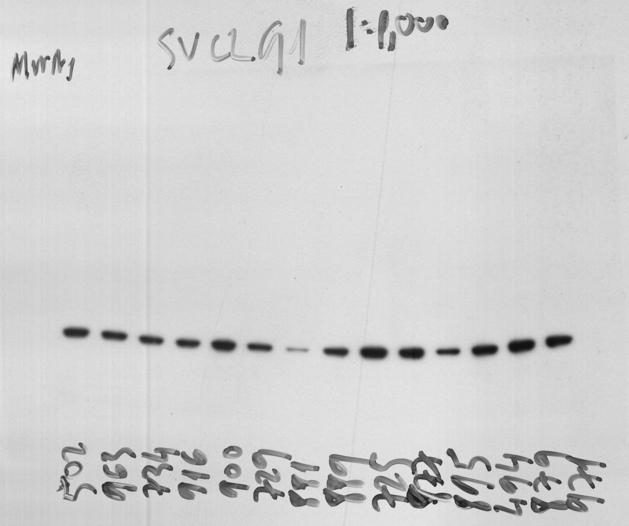
SVCL91
1:1,000

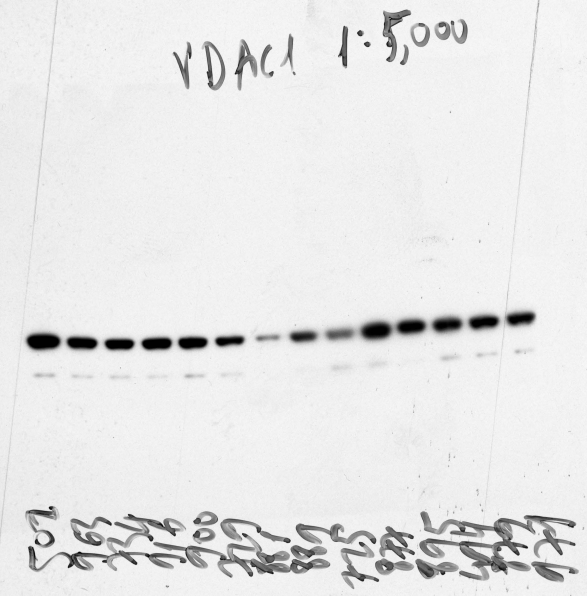
VDAC1 1:5,000

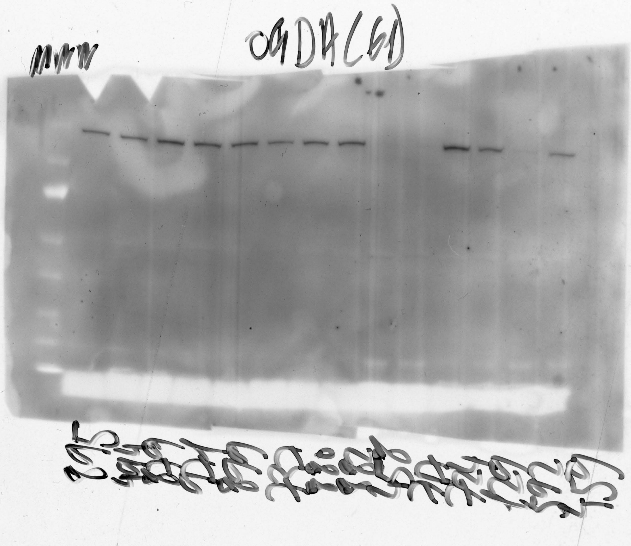

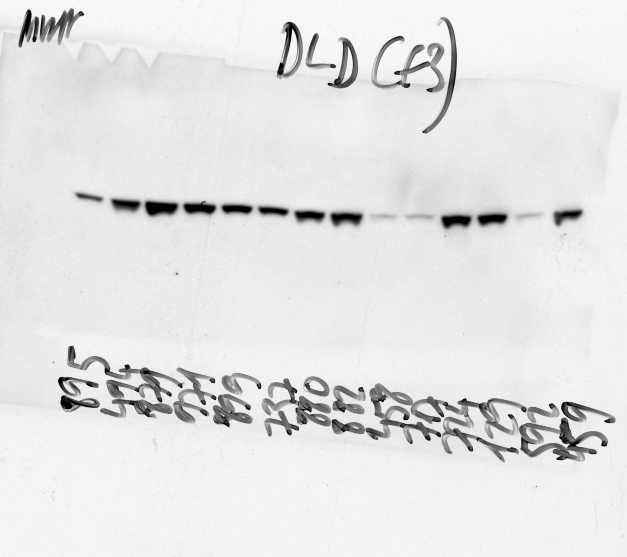
DLD (F3)

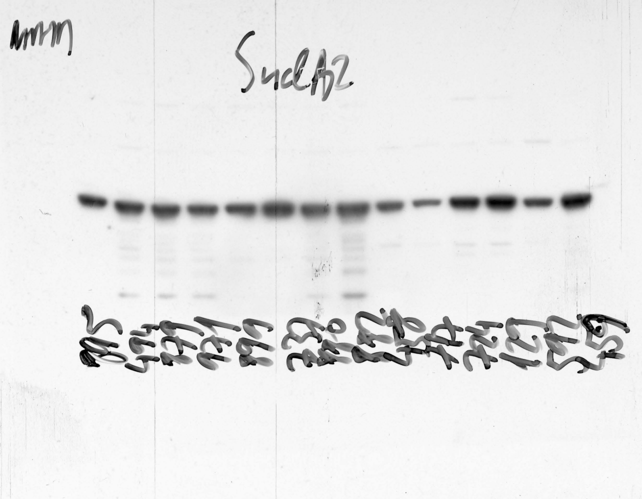
SuclA2

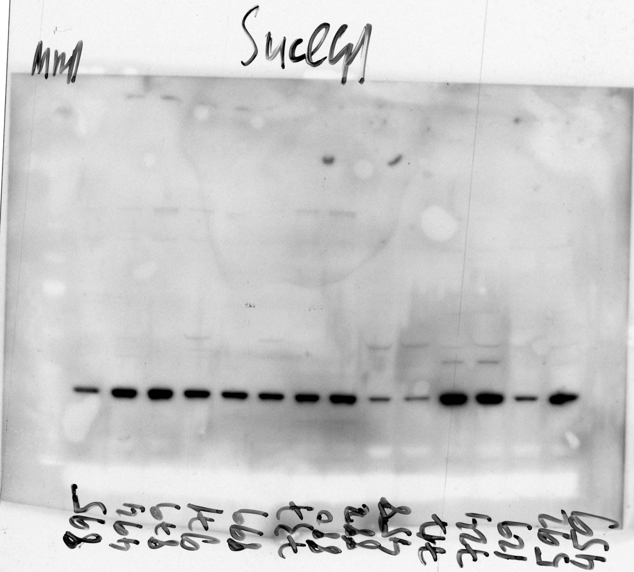

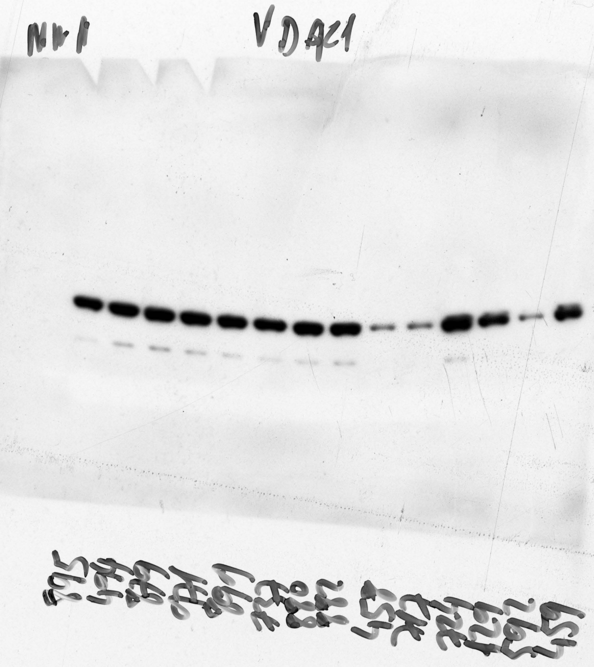
VDAC1

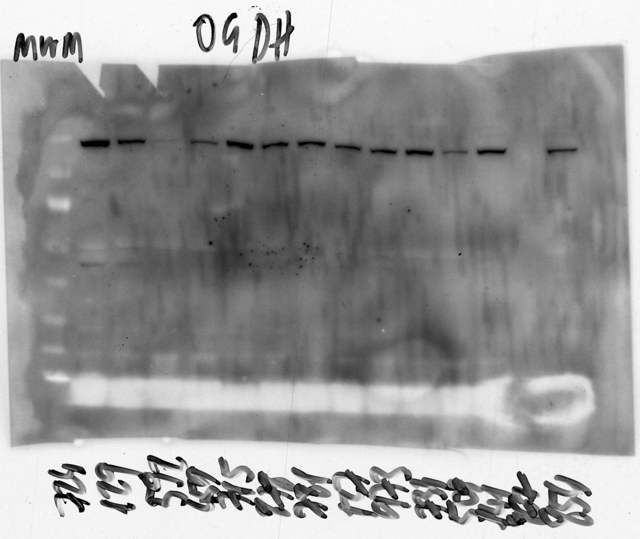
MWM
OGDH

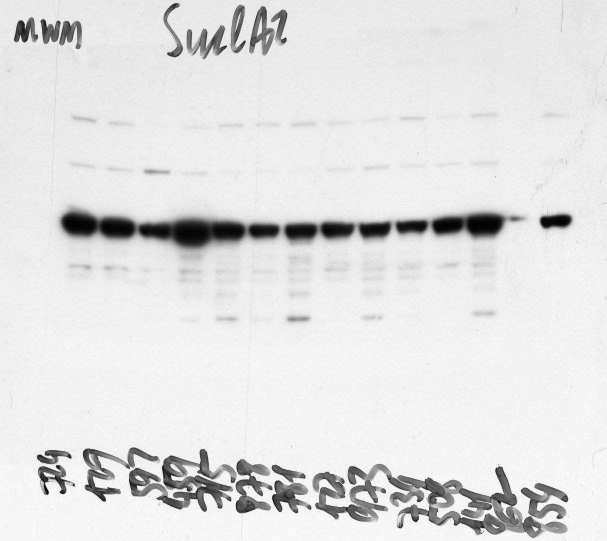
MWM

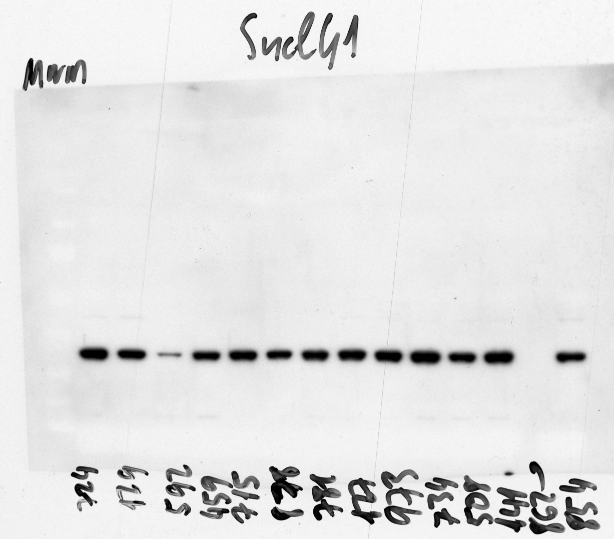
SuclG1

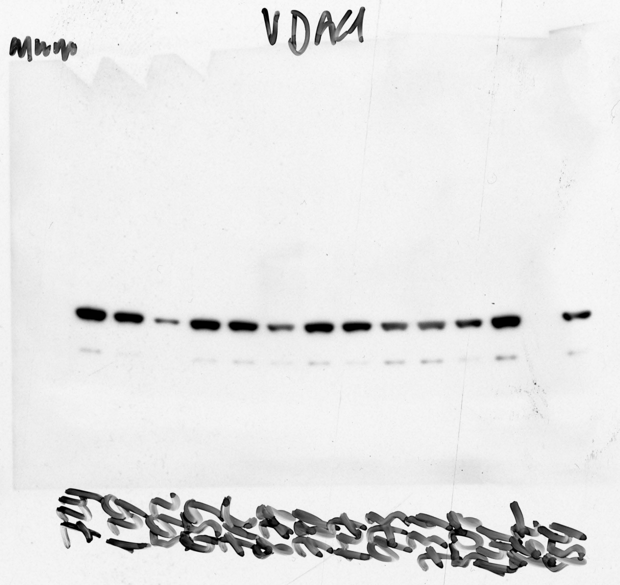
VDAC1

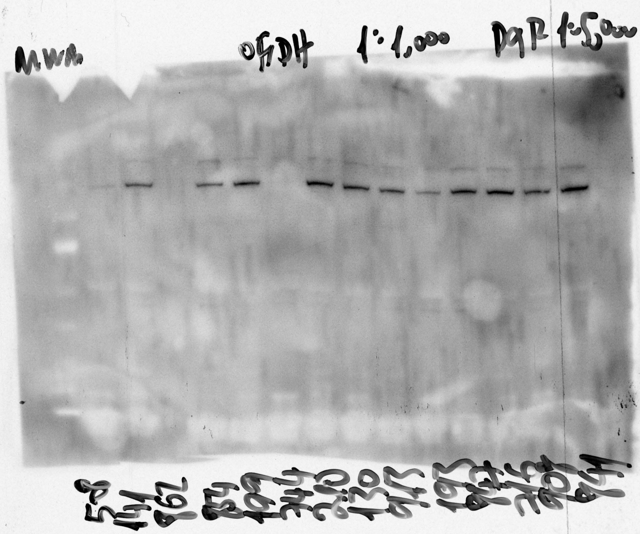
1:1,000
1:5,000

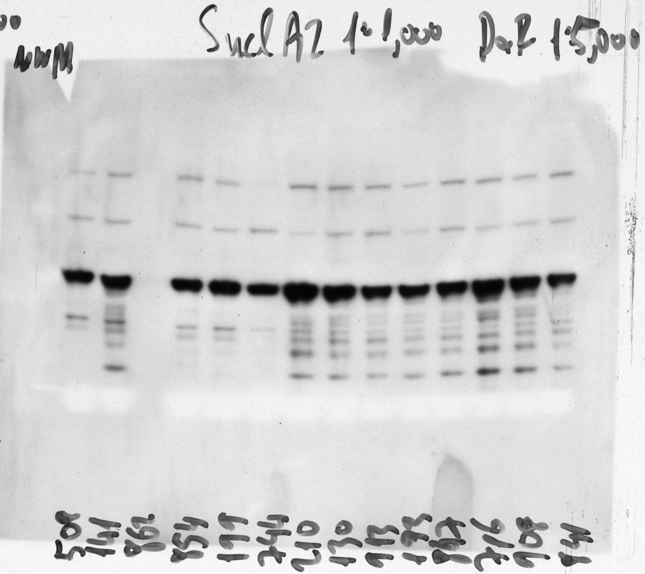
SucIA2 1:1,000
DaR 1:5,000
MWM

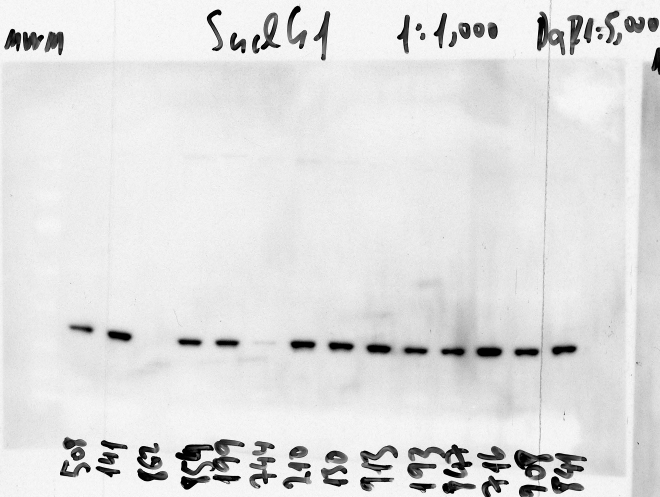
SucLG1
1:1,000

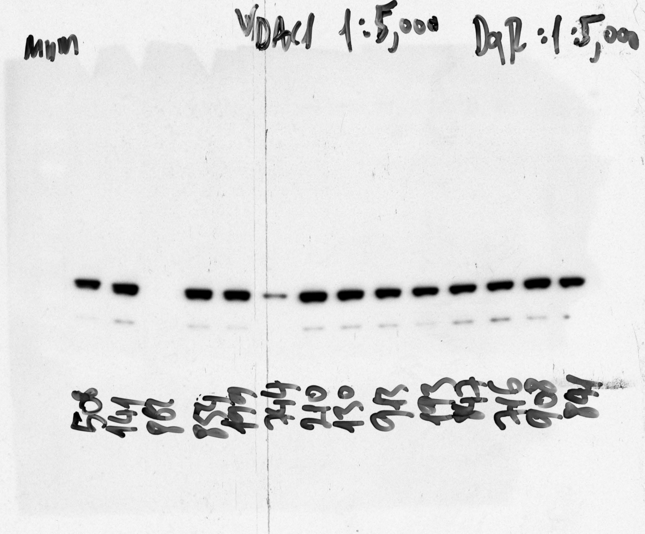
VDAC1 1:5,000

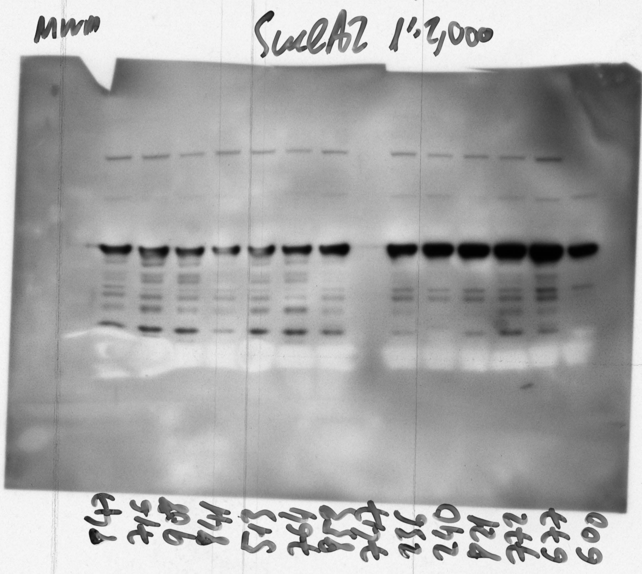
MWM
SucLA2 1:2,000

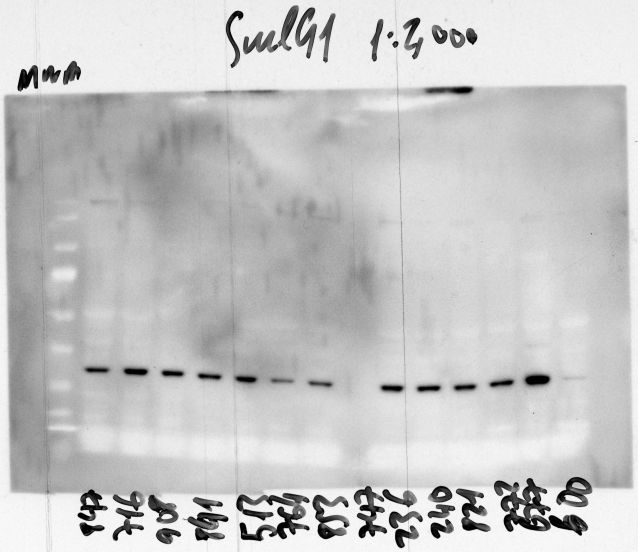
SmlG1 1:3,000
MWM

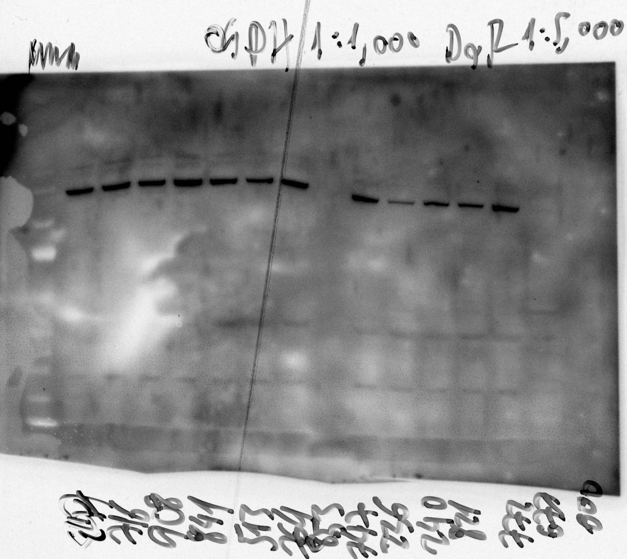
GAPDH 1:1,000

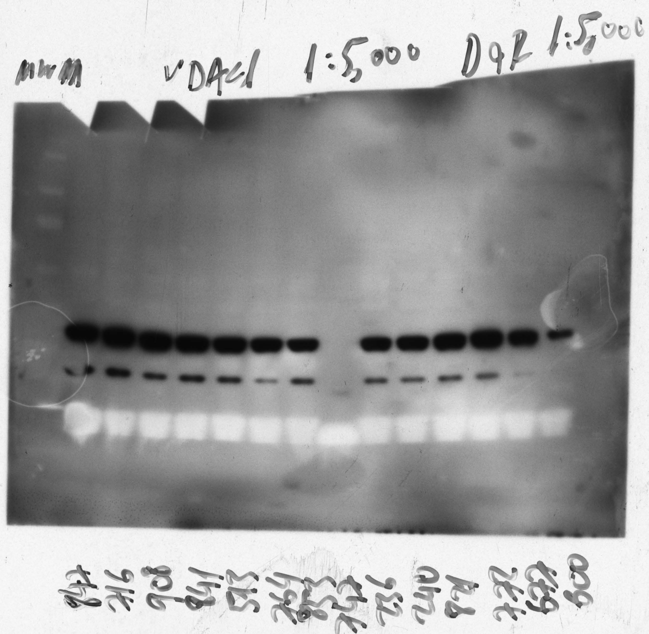
MWM
VDAC1
1:5,000
1:5,000
