# Supplementary material for: Exclusive neuronal detection of KGDHC-specific subunits in the adult human brain cortex despite pancellular protein lysine succinylation
Source: Brain Struct Funct. 2020 Jan 25;225(2):639–67. doi: 10.1007/s00429-020-02026-5 (PMC7046601; doi:10.1007/s00429-020-02026-5)
Supplement: Supplementary file 2 — Supplementary file2 (DOCX 12 kb) [file 429_2020_2026_MOESM2_ESM.docx]

**SUPPLEMENTAL MATERIAL**

Exclusive neuronal detection of KGDHC-specific subunits in the adult human brain cortex despite pancellular protein lysine succinylation

Arpad Dobolyi^1,2^, Attila Bago^3^, Miklos Palkovits^1^, Natalia S Nemeria^4^, Frank Jordan^4^, Judit Doczi^5^, Attila Ambrus^5,6^, Vera Adam-Vizi^5,6^ and Christos Chinopoulos^5^

^1^MTA-ELTE Laboratory of Molecular and Systems Neurobiology, Department of Physiology and Neurobiology, Hungarian Academy of Sciences and Eotvos Lorand University, Budapest,1117, Hungary

^2^Department of Anatomy, Histology and Embryology, Semmelweis University, Budapest, 1094, Hungary

^3^National Institute of Neurosurgery, Budapest, 1145, Hungary

^4^Department of Chemistry, Rutgers University, Newark, NJ, 07102-1811, USA

^5^Department of Medical Biochemistry, Semmelweis University, Budapest, 1094, Hungary

^6^MTA-SE Laboratory for Neurobiochemistry, Semmelweis University, Budapest, 1094, Hungary

Running title: KGDH in human brain

To whom correspondence should be addressed: Christos Chinopoulos, MD, PhD, Department of Medical Biochemistry, Semmelweis University, Budapest, Tuzolto st. 37-47, 1094, Hungary, Tel: +361 4591500 ext. 60024; Fax: +361 2670031; E-mail: [chinopoulos.christos@eok.sote.hu](mailto:chinopoulos.christos@eok.sote.hu)

Legend to supplemental figure 1: Scanned images of whole blots shown in figure 9 of the main text.
